# Characterization of long and stable *de novo* single alpha-helix domains provides novel insight into their stability

**DOI:** 10.1038/srep44341

**Published:** 2017-03-13

**Authors:** Marcin Wolny, Matthew Batchelor, Gail J. Bartlett, Emily G. Baker, Marta Kurzawa, Peter J. Knight, Lorna Dougan, Derek N. Woolfson, Emanuele Paci, Michelle Peckham

**Affiliations:** 1 Astbury Centre for Structural Molecular Biology, Faculty of Biological Sciences, University of Leeds, Leeds, LS2 9JT, UK; 2 School of Chemistry, University of Bristol, Cantock’s Close, Bristol, BS8 1TS, UK; 3 Astbury Centre for Structural Molecular Biology and School of Physics and Astronomy, University of Leeds, Leeds, LS2 9JT, UK; 4 School of Biochemistry, University of Bristol, Biomedical Sciences Building, Bristol, BS8 1TD, UK; 5 BrisSynBio, University of Bristol, Life Sciences Building, Bristol, BS8 1TQ, UK

## Abstract

Naturally-occurring single α-helices (SAHs), are rich in Arg (R), Glu (E) and Lys (K) residues, and stabilized by multiple salt bridges. Understanding how salt bridges promote their stability is challenging as SAHs are long and their sequences highly variable. Thus, we designed and tested simple *de novo* 98-residue polypeptides containing 7-residue repeats (AEEEXXX, where X is K or R) expected to promote salt-bridge formation between Glu and Lys/Arg. Lys-rich sequences (EK3 (AEEEKKK) and EK2R1 (AEEEKRK)) both form SAHs, of which EK2R1 is more helical and thermo-stable suggesting Arg increases stability. Substituting Lys with Arg (or *vice versa)* in the naturally-occurring myosin-6 SAH similarly increased (or decreased) its stability. However, Arg-rich *de novo* sequences (ER3 (AEEERRR) and EK1R2 (AEEEKRR)) aggregated. Combining a PDB analysis with molecular modelling provides a rational explanation, demonstrating that Glu and Arg form salt bridges more commonly, utilize a wider range of rotamer conformations, and are more dynamic than Glu–Lys. This promiscuous nature of Arg helps explain the increased propensity of *de novo* Arg-rich SAHs to aggregate. Importantly, the specific K:R ratio is likely to be important in determining helical stability in *de novo* and naturally-occurring polypeptides, giving new insight into how single α-helices are stabilized.

Naturally occurring single α-helices (SAHs) are found in ~4% of proteins^1^,^2^,^3^, and have been commonly misidentified as coiled coils^4^. SAHs are not stabilized by a tertiary fold, remain monomeric and highly helical over a broad range of salt concentrations and pH, and exhibit a thermal denaturation profile that is only weakly cooperative^2^,^5^. They are stiff enough to replace the canonical lever in myosin^6^, can behave as a “constant force spring” when extended by small (<30 pN) forces^5^, and have a persistence length of ~15 nm (equivalent to ~100 residues of α-helix)^7^. Many putative SAHs have now been identified in a wide range of proteins with diverse functions, typically ranging in length between 40 and ~200 residues^1^,^2^,^7^,^8^,^9^. However, only a few of these have been characterized experimentally and in detail^4^,^5^,^6^,^9^,^10^,^11^,^12^. Most recently, it has been shown that in some cases, a region of the SAH can mediate binding to other proteins^13^,^14^. Thus it is important to understand the underlying stability of these intriguing domains, in order to understand their contribution to protein function.

SAHs are likely to form when a sequence is rich in acidic and basic residues (Glu, E; Arg, R and Lys, K), lacks a hydrophobic seam, and contains many potential intrahelical interactions (salt bridges) between either E and K (E–K), or E and R (E–R). These pairings are spaced three or four residues apart (*e.g*. “E → K(+3)” or “K → E(+3)” where the K residue is 3 residues downstream or upstream from E respectively)^2^,^4^. However, understanding how such salt bridges promote the highly helical states of natural sequences is challenging, as SAHs have a range of different potential salt bridges in terms of sequence separation and residue type. Single E–K or E–R pairs of this type are known to promote the folding of monomeric α-helices in short (<20 residue) alanine-based peptides^15^,^16^, and thus are assumed to stabilize long SAHs. Studies on short peptides have additionally suggested that K → E(+4) pairs are more helix-stabilizing than the reversed E → K(+4) orientation^17^, resolving previous conflicting reports^16^,^18^,^19^. However, the equivalent study for E–R pairs is lacking. Although it was recently reported that E–R pairs may be more stabilizing than E–K in very short (<12 residue) peptides^20^, it is unclear which pairings are utilized, or why E–R pairs are more stabilizing.

To determine how Lys and Arg contribute to the stability of long SAHs, we first designed, expressed and characterized *de novo* SAHs. These long (98-residue) polypeptides contain 7-residue repeats, AEEEXXX, where X is K or R, and were designed to emulate the properties of SAHs using simpler constructs. Their length was specifically chosen to be similar to that of many natural SAHs^2^ as a better test of the properties of a SAH compared to short peptides. To interpret our experimental findings, we analysed side chain interactions in α-helices from the Protein Data Bank (PDB)^21^ for E–R pairs and compared these to E–K pairs. We additionally performed molecular dynamics (MD) simulations to investigate salt-bridge formation and the dynamic behaviour of salt bridges formed by E–K and E–R parings in the SAHs. Re-engineered SAHs from myosin-6, in which all Lys residues were replaced with Arg, and *vice versa*, showed behaviours consistent with those for the *de novo* polypeptides and computational analysis. We find that Lys and Arg are not completely interchangeable, but make distinct and significant contributions to the stability of SAHs. Our findings also provide clear design rules for generating or re-engineering these domains.

## Results

### SAHs by design: EK3 and ER3 *de novo* polypeptides

**Figure 1:**
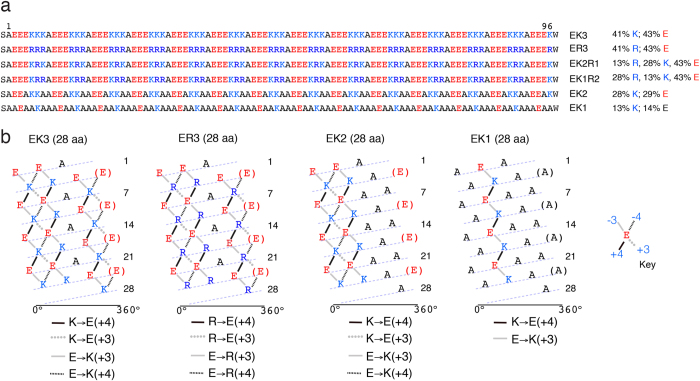
Sequence design for *de novo* polypeptides used in this study. (**a**) The sequences for each of the *de novo* polypeptides tested (each contain 98 residues including the N-terminal Ser (S) and C-terminal Trp (W)). Each *de novo* polypeptide was expressed and purified from *E. coli*. The percentages of Glu, Arg and Lys residues are shown for each *de novo* polypeptide. (**b**) Helical net plots^2^ showing 28 residues (4 of the 7 residue repeats) of the EK3, ER3, EK2 and EK1 sequences. In these plots, the helix has been ‘cut’ along a helical track and unwound so that it can be displayed in 2-D. The dashed line marks the path of the polypeptide chain. Residues along the cut position at the far left are repeated in parentheses on the right so all potential interactions can be shown. The key for the different salt bridge interactions are shown beneath, where the traditional terminology is changed for ease of reading, *e.g*. K_i_ → E_i+4_ is replaced by K → E(+4).

To test the relative contributions of Lys and Arg to SAHs, we designed *de novo* polypeptides that contained either E–K or E–R pairs termed EK3 and ER3 (Fig. 1), both of which were expected to behave as SAHs. To match the 3.6 residues per turn of the α-helix as closely as possible, the *de novo* polypeptides were based on the 7-residue repeats, AEEEKKK and AEEERRR, respectively (Fig. 1a). These maximize the possibilities for each Glu and Lys (or Arg) residue to satisfy the favoured downstream and upstream salt bridge partner suggested by previous modelling^7^. There are three E → K(+3) or E → R(+3) potential pairs within each repeat, and three K → E(+4) or R → E(+4) pairs between successive repeats (Fig. 1b), which effectively saturates the possible stabilizing interactions. Two alternative E → K(+4) or E → R(+4) pairs are also possible within each repeat, as well as two K → E(+3) or R → E(+3) pairs between successive repeats. Single alanine residues were chosen as ‘fillers’ in the repeats to avoid potential repulsive and helix-destabilizing interactions between like-charged residues (E–E and K–K or R–R) along the helix. Alanine has been used extensively in short helical peptides^22^,^23^, as it maintains overall charge neutrality, does not affect neighbouring charge–charge interactions, and has a high helix-propensity^24^. In addition, alanine is commonly found in known SAHs^9^, including those in caldesmon^25^, and the Kelch-motif family protein^10^.

**Figure 2:**
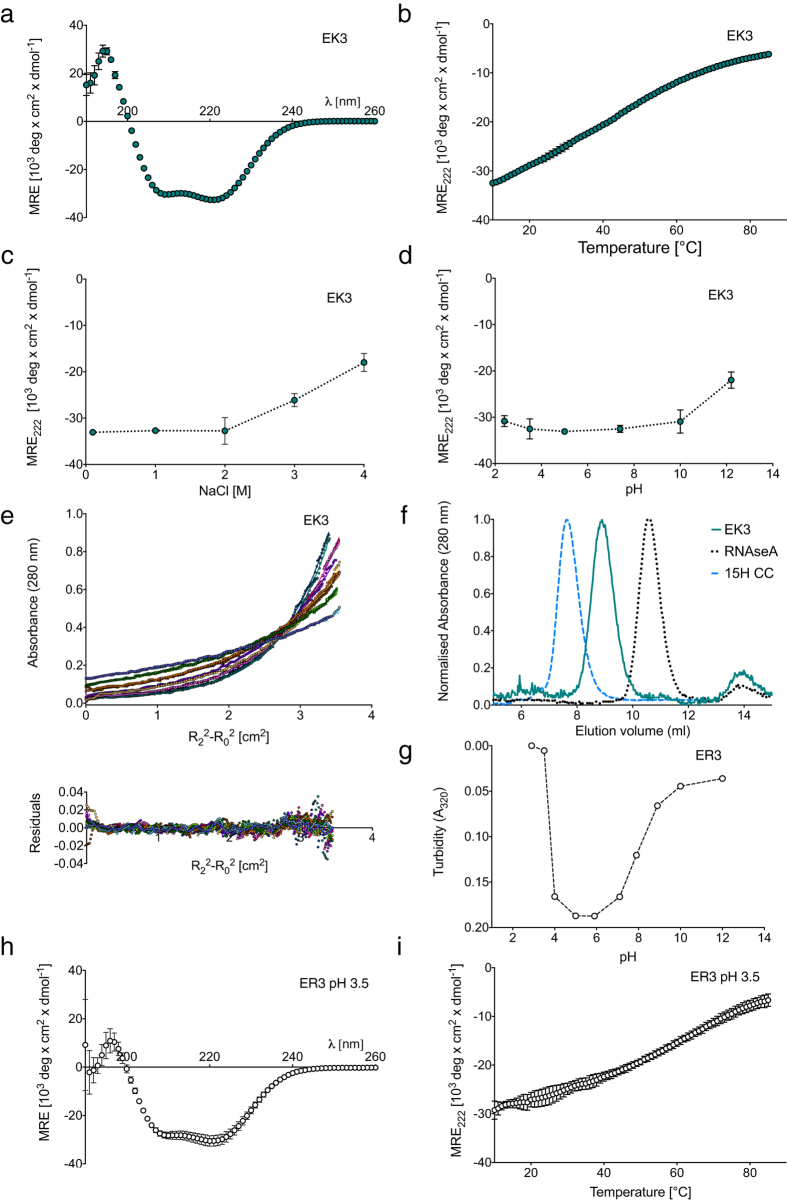
Properties of EK3 and ER3. (**a**) CD spectrum (pH 7.4, 10 °C, 10–20 μM *de novo* polypeptide in 100 mM NaCl) of EK3. (**b**) Thermal denaturation of EK3. The mean residue ellipticity (MRE) at 222 nm is shown as a function of temperature (10–85 °C, pH 7.4, 100 mM NaCl). (**c**) MRE at 222 nm as a function of salt concentration (pH 7.4, 10 °C), and (**d**) MRE at 222 nm as a function of pH (100 mM NaCl, 10 °C). Mean (±S.D.) values are plotted. (**e**) AUC data for EK3 showing the data (circles) and single ideal species fits (solid lines), together with the residuals of the fits. Each colour corresponds to a different speed. The fit to the AUC data for EK3 suggests a mass of 13.3 kDa, 1.13 × that predicted (11.8 kDa). (**f**) Results for size exclusion chromatography of EK3. For comparison, the results for a coiled-coil protein (15 heptad construct from β-cardiac myosin-2 tail: 15 H CC, with a dimer mass of 25 kDa) and a globular protein with a similar molecular mass (ribonuclease A/RNAse A, 13.7 kDa) are also shown. (**g**) Solubility of ER3 (15 μM in 100 mM NaCl) over a range of pH, as measured by turbidity measurements at 320 nm. (**h**) CD spectrum (pH 3.5, 10 °C, 10–20 μM *de novo* protein in 100 mM NaCl) of ER3. (**i**) Thermal denaturation of ER3 at pH 3.5. Conditions are the same as in (**h**).

The expressed and purified EK3 polypeptide exhibited the behaviour expected for a SAH. It was highly helical (>90% at 10 °C, 17 μM in 100 mM NaCl, Fig. 2a) and unfolded upon heating with a broad, non-cooperative transition (Fig. 2b), as shown by circular dichroism (CD) spectroscopy experiments. EK3 helicity remained high at high salt concentration (its helicity in 4 M NaCl was 60% of that in 100 mM NaCl (Fig. 2c)), and over a wide pH range (pH 2 to pH 10; Fig. 2d). EK3 is monomeric as shown by analytical ultracentrifugation (AUC) (Fig. 2e), and has an elongated shape as shown by size exclusion chromatography (SEC), where it eluted faster than a globular protein (ribonuclease A) of similar mass but slower than a dimeric coiled coil (15 H CC; ref. 5), which is elongated and has approximately double the mass of EK3 (Fig. 2f).

Surprisingly, the purified ER3 polypeptide did not behave as a SAH. It aggregated at low salt and neutral pH (100 mM NaCl, pH 7.4), as evidenced by the high turbidity of the solution (Fig. 2g) and was only soluble at low pH (<3.5). As a high proportion of the Glu residues will be protonated at low pH, ER3 is expected to have a net positive charge that allows it to be soluble. Although ER3 was highly helical (Fig. 2h) and had similar melting behaviour at pH 3.5 (Fig. 2i) to EK3 at pH 7.4 (Fig. 2b), we concluded that its lack of solubility at neutral pH was inconsistent with that of a SAH.

**Figure 3:**
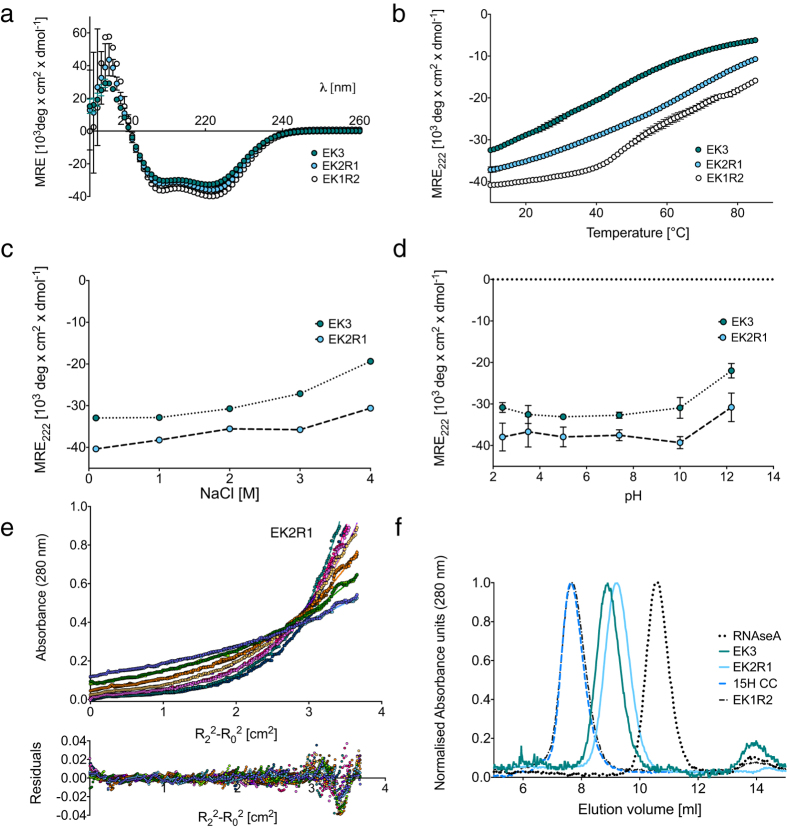
Properties of EK2R1 and EK1R2. (**a**) CD spectra (pH 7.4, 10 °C, 10–20 μM *de novo* polypeptide, 100 mM NaCl) for EK2R1 (light green) and EK1R2 (open circles). The data from Fig. 2 for EK3 (green) is repeated here to enable comparison. (plots show mean ± S.D.) (**b**) Thermal denaturation of EK3, EK2R1 and EK1R2. The MRE_222_ values at each temperature are shown (mean ± S.D.). Conditions as in (**a**). The profile for EK1R2 exhibits greater cooperativity than EK3. (**c**) MRE at 222 nm as a function of salt concentration (pH 7.4, 10 °C) for EK3 and EK2R1. Mean MRE_222_ values are plotted (±S.D.) (**d**) MRE at 222 nm as a function of pH (100 mM NaCl, 10 °C). Mean MRE_222_ values are plotted (±S.D.). (**e**) AUC data for EK2R1 showing the data (circles) and single ideal species fits (solid lines), together with the residuals of the fits. Each colour corresponds to a different speed. The fit to the AUC data for EK2R1 indicates a mass of 12.7 kDa, 1.04× that predicted (12.2 kDa). (**f**) Results for size exclusion chromatography of EK2R1 and EK1R2. For comparison, the results for EK3, a coiled-coil protein (15 heptad construct from β-cardiac myosin-2: 15 H CC) and a globular protein with a similar molecular mass (ribonuclease A/RNAse A, 13.7 kDa) are also shown.

To further test the contribution of Arg to SAHs, we expressed and tested two additional *de novo* polypeptides, EK2R1 (AEEEKRK) and EK1R2 (AEEEKRR), in which either one or two Lys residues per repeat in EK3 were replaced by Arg (Fig. 1a). Replacing one Lys residue with Arg (EK2R1) resulted in a peptide that behaved as a SAH that was more helical (Fig. 3a) and thermally stable (Fig. 3b) compared to EK3. It was also more helical at high salt (4 M NaCl) than EK3 (Fig. 3c), and remained helical over a range of pH (Fig. 3d). AUC confirmed that EK2R1 was monomeric (Fig. 3e) and SEC showed it to be elongated but not oligomerized (Fig. 3f). In contrast, while replacing two Lys residues with Arg (EK1R2) resulted in a peptide that was soluble at pH 7.4 and highly helical (Fig. 3a), its more complex thermal unfolding behaviour suggested that it was not monomeric (Fig. 3b). Both AUC (Supplementary Fig. S1a) and SEC (Fig. 3f) showed that it was indeed oligomeric. Therefore, while EK2R1 behaves as a SAH, EK1R2 does not.

To explore the sequence requirements for *de novo* SAH formation, we designed two further polypeptides, EK1 and EK2 (Fig. 1). These had lower proportions of charged residues, and concomitant increases in helix-favouring alanine residues. EK2, which contained the repeat AEEAKKA (Fig. 1a), was highly helical (Supplementary Fig. S1b) but apparently dimerized. Its high helicity persisted up to 85 °C (Supplementary Fig. S1c) and in SEC, EK2 eluted between two known dimeric coiled-coil proteins of 25 kDa and 18.3 kDa (dimer mass, Supplementary Fig. S1d). However, an accurate molecular mass by AUC could not be determined due to protein precipitation (Supplementary Fig. S1e). We propose that the alanine residues of EK2 probably form a hydrophobic seam to promote dimerization into a coiled coil. EK1, which has AAEAAKA repeats (Fig. 1a), was soluble only in a very low ionic strength buffer (10 mM NaCl, 5 mM Tris, pH 7.4; Supplementary Fig. S1h). Under these conditions EK1 was helical (Supplementary Fig. S1f) but the thermal melt profiles indicated that it formed oligomeric species (Supplementary Fig. S1g). Thus, reducing the proportions of charged residues in this manner to form a polyalanine based construct is not compatible with the formation of a long SAH.

### Re-engineering natural SAHs predictably alters their properties

**Figure 4:**
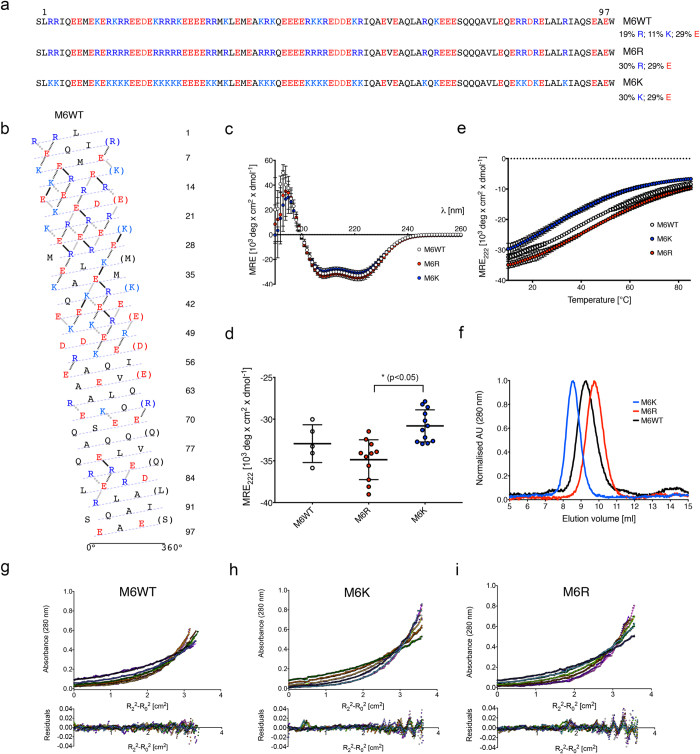
Properties of re-engineered myosin-6 SAHs. (**a**) Sequences of the proteins M6WT, M6R and M6K, together with the percentage of Arg, Lys and Glu content. (**b**) Helical net plot^2^ for the M6WT SAH. (**c**) CD spectra (pH 7.4, 10 °C, 10–20 μM protein, 100 mM NaCl) for each of the three proteins. (**d**) Mean values (±S.D.) of repeat measurements for MRE_222_ values at 10 °C. The mean values for M6R and M6K were significantly different (p < 0.05). (**e**) Thermal denaturation. The MRE_222_ values at each temperature are shown. (**f**) SEC of each of the three proteins. AUC data for (**g**) M6WT, (**h**) M6K and (**i**) M6R. Presentation as per Fig. 2e. The fits to the data indicate a mass of 12.8 kDa for M6WT: (1.02 × calculated monomer mass of 12.5 kDa); 12.6 kDa for M6K (1.05 × calculated monomer mass of 12.0 kDa) and 12.5 kDa for M6R (0.98 × monomer mass of 12.8 kDa).

The data above show that replacing a single Lys per repeat with Arg (EK3 to EK2R1) increases both the helicity and resistance to thermal unfolding. To test if this translates to a natural SAH, we substituted all the Lys residues in the SAH of myosin-6 with Arg (M6WT to M6R, Fig. 4a,b, which increases % Arg content from 19 to 30%), and, in a second construct, all of the Arg residues with Lys (M6K, Fig. 4a, which increases %K content from 11 to 30%). M6R was slightly more helical compared to M6WT, while M6K was slightly less helical (Fig. 4c), and M6R was significantly more helical than M6K (Fig. 4d). Similarly, the apparent order of thermal stability was M6R > M6WT > M6K (Fig. 4e). In SEC experiments M6R, M6WT and M6K eluted similarly, although the elution time increased with increasing Arg content (Fig. 4f) despite M6R having a larger mass and being more helical. The slower elution could arise from a smaller hydration shell for M6R compared to M6WT and M6K, or from stronger interactions of Arg with the column. M6WT, M6K and M6R were all found to be monomeric by AUC (Fig. 4g–i). Thus, modulating K/R content in a natural SAH has similar effects on helicity and thermal behaviour as discovered for the *de novo* SAHs, EK3 and EK2R1. Interestingly, increasing the Arg content in this case did not promote aggregation. This may be due to the non-repetitive nature of the sequence compared to *de novo* polypeptides, and more diverse content of amino acids other than E, K and R.

### E–R and E–K pairings have different properties in protein X-ray crystal structures

The data presented above show that the AEEEXXX repeat pattern is suitable for designing model SAHs, with X = R increasing stability, albeit in small doses, and X = K important for solubility. What gives rise to the increased stability with respect to thermal unfolding in Arg-containing sequences? We addressed this by analysing salt bridges in α-helices of the PDB, and by MD simulations of our *de novo* polypeptide designs. Throughout, a salt bridge was assigned for an E–R (or E–K) pair in a helix if the centroid of Glu Oε1 and Oε2 atoms was <4 Å from any of the Nε, NH1 or NH2 atoms in Arg (or from the Nζ atom in Lys).

The PDB analysis showed that E–R pairs were more frequent than expected by chance from the joint probabilities of individual amino acids (Supplementary Table S1), as previously described for E–K^17^. Overall, the total number of E–R pairs are similar to those reported for E–K pairs (see Table S8 in ref. 17). The preference for E–R pairs occurred in the order E → R(+3) > R → E(+4) > E → R(+4) > R → E(+3) (observed/expected in Supplementary Table S1). In contrast, E–K pairs^17^, showed a preference for + 4 pairs over + 3 pairs in the order K → E(+4) > E → K(+4) > K → E(+3) ≈ E → K(+3).

**Figure 5:**
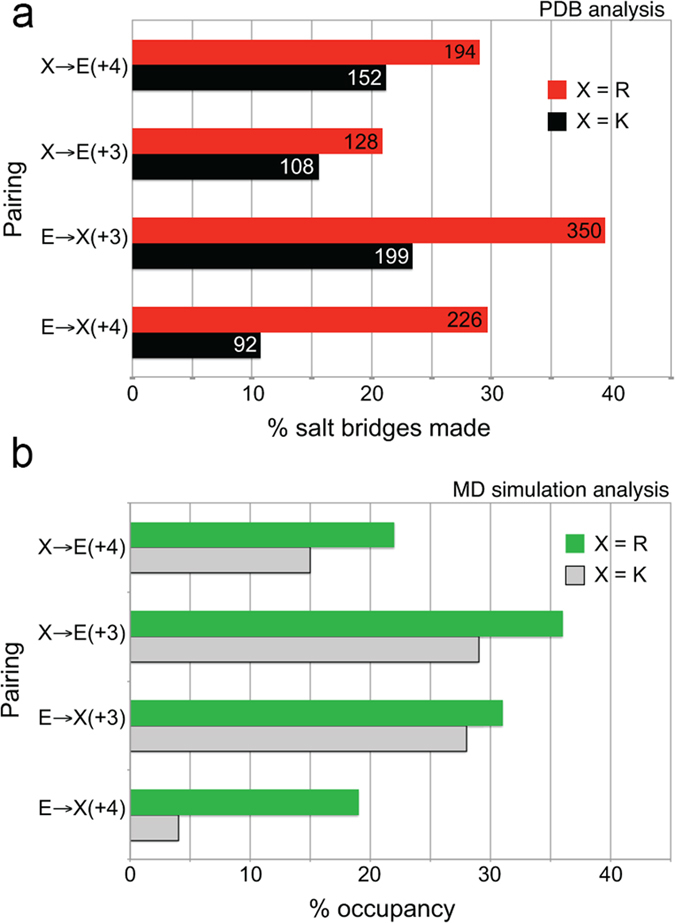
Structural analysis of helices in the PDB, and equilibrium properties at 300 K from MD simulations. (**a**) PDB analysis. For each pairing, the number of salt bridges observed in helices in the PDB is shown on the bar. The bar length represents this number of salt bridges observed as a percentage of pairings identified from protein sequence (see Supplementary Table S1). The values for E–K pairings are taken from Baker *et al*.^17^. (**b**) MD simulation analysis. For each pairing mode, the bar length represents the occupancy of salt bridges observed as a percentage of all pairings from simulations of EK3, EK2R1, EK1R2 and ER3.

The analysis also showed that E–R pairs form salt bridges more frequently than E–K pairs (Fig. 5a, Supplementary Table S1). For example, 40% of E → R(+3) pairings formed salt bridges, compared to 23% of E → K(+3) pairings. Similar trends were observed for the remaining three types of pairings. E → R(+3) pairs, being the most over-represented and the most likely to actually form salt bridges, are likely to be the most stabilizing of the four E–R pairing options. This contrasts with E–K pairs, where K → E(+4) is thought to be the most stabilizing^17^.

Several specific side-chain rotamer conformations predominate for Arg and Glu in salt bridges. For individual amino acids (black bars in Supplementary Fig. S2), Arg has two preferred rotamer (*χ*_1_, *χ*_2_) combinations, *g*^−^*t* (38%) and *tt* (25%), and two minor conformers (*tg*^+^, 10% and *g*^−^*g*^−^, 5%). These two major Arg rotamers are the same as those found previously for Lys: *g*^−^*t* (39%) and *tt* (32%)^17^. Glu has three preferred *χ*_1_, *χ*_2_ combinations, *g*^−^*t* (36%), *tt* (25%) and *g*^−^*g*^−^ (19%). In E → R(+3) salt bridges, the *tg*^+^/*tt* (E/R) combination (Supplementary Table S2) was the most prevalent. This combination has the second most-preferred conformation for Arg, and a disfavoured conformation for Glu (Supplementary Fig. S2). E → R(+3) salt bridges also utilized other rotamer combinations *g*^−^*g*^−^/*tt, g*^−^*g*^−^/*g*^−^*t* and *tg*^+^/*g*^−^*t* (21%, 14% and 14%, respectively, Supplementary Table S2), which mostly draw on the two major Arg conformations and the more-preferred Glu conformation *g*^−^*g*^−^. Overall, the majority of the E → R(+3) pairings had Glu in a less favoured conformation. The entropic penalty that this incurs is likely to be offset by the use of more-favourable Arg conformations and the multiple modes available to make profitable salt bridges.

Rotamer combinations utilized by E–R pairs to form salt bridges were less dominated by a single combination, in contrast to E–K pairs^17^. The frequency of the favoured *tg*^+^/*tt* rotamer combination in E → R(+3) (47%) was lower than the dominant combination (*g*^−^*g*^−^/*g*^−^*t*) for E → K(+3) (77%, Supplementary Table S2). Similar reductions in the predominance of a single rotamer combination were also found for the E → R(+4) and R → E(+4) pairs (Supplementary Table S2, Supplementary Fig. S2). The contribution of *tt/g*^−^*t* reduced from 68% in E → K(+4) to 34% in E → R(+4); and 75% in K → E(+4) to 56% in R → E(+4) for *g*^−^*t/tt*. R → E(+3) and K → E(+3) salt bridges were spread in similar fashion across four rotamer combinations without one particularly dominant contributor.

Taken together, the PDB analysis demonstrates that E–R pairs are more prevalent and display a greater range of rotamer conformations than E–K pairs to make salt bridges. The increased number of salt bridges made by E–R pairs is probably related to the Arg side chain being longer and the multi-dentate guanidinium group having more possibilities of interactions compared with the amino side chain of lysine. The increased variability in their rotamer conformations suggests that the E–R pairs are more dynamic than E–K, whilst still productively engaging in salt-bridge interactions. Indeed, structural superpositions of helices containing salt bridges (Supplementary Fig. S3) revealed significant variation in Glu and Arg side chain conformations for each of the four E–R arrangements.

### Molecular dynamics simulations show different dynamics for E–K and E–R salt bridges

**Figure 6:**
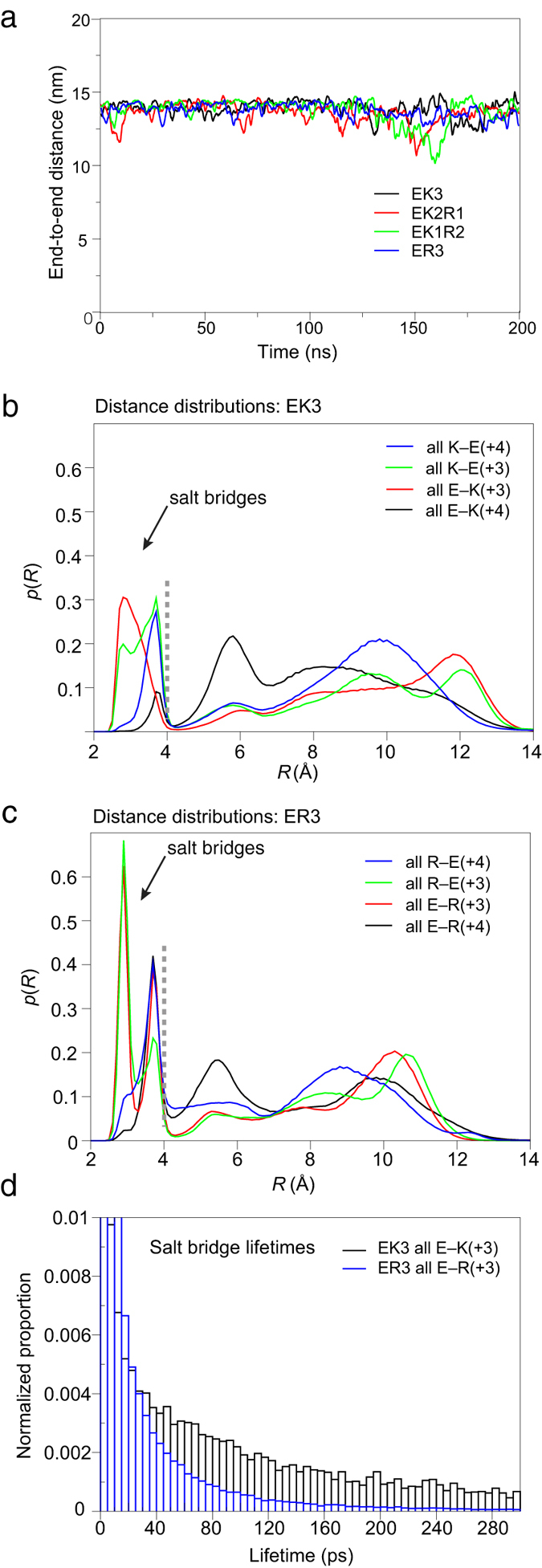
Characteristics of *de novo* proteins during MD simulations. (**a**) Plots of the end-to-end distance for each protein during the simulation. (**b**) Normalized distance distributions in EK3 for all K → E(+4), K → E(+3), E → K(+3) and E → K(+4) pairings. Pairing distances are between the centre-of-mass (CoM) of the Glu Oε atoms and the Nζ atom in Lys. The plots were generated from histograms of all salt bridge distances for pairings of each type using 0.1 Å bins. The grey dashed line at 4 Å represents the cut-off point for considering a pairing as a salt bridge. Conformations with distances up to 4 Å (*i.e*. to the left side of this line) are considered to have formed a salt bridge. The peaks at 5.6–6.0 Å can be attributed to ‘indirect’ salt bridges separated by one water molecule, which are not included in the analysis^46^. (**c**) Normalized distance distributions in ER3 for all R → E(+4), R → E(+3), E → R(+3) and E → R(+4) pairings. The pairing distance is the smallest of the distances between the CoM of the Glu Oε atoms and each of the three side chain N atoms in Arg. (**d**) A normalized histogram plot of salt bridge lifetimes (5 ps bins), for all E → K(+3) and E → R(+3) salt bridges in EK3 and ER3, respectively.

To investigate the dynamic behaviour of E–K and E–R salt bridges, we performed MD simulations. The *de novo* polypeptides all remained as near-complete continuous α-helices (≥95% helix), which were elongated (not bundled) throughout the 200 ns simulations (Fig. 6a). Clear transitions between states in which the side chains for residues in E–K or E–R pairs were either in close proximity (*i.e*., forming a salt bridge) or well-separated (non-interacting) were observed during the simulations, as illustrated by the trajectory for K49 in EK3 (Supplementary Fig. S4). Calculating the distances between side chains in all available pairings throughout the simulation trajectories, showed salt bridge occurrence as distinct peaks in probability below 4 Å (Fig. 6b and c, Supplementary Fig. S5). This agrees well with the cut-off for salt bridge assignment of 4 Å used in the PDB database interrogation. Peaks observed at ~2.8 Å and ~3.7 Å arise from salt bridges that utilize different rotamer combinations.

MD simulations showed E–K salt bridges were less highly occupied than E–R (Fig. 5b, Supplementary Table S3). K → E(+4) pairs were more likely to form salt bridges than E → K(+4) pairs (Fig. 5b, Supplementary Table S3) as previously shown experimentally^17^, and in good agreement with the PDB analysis (Fig. 5a). The % salt bridge occupancy for R → E(+4) and E → R(+4) pairs was more similar (Fig. 5b, Supplementary Table S3), also in agreement with the PDB analysis (Fig. 5a). The only difference in trends we observed between the PDB results and the MD simulations is that the occupancy of R → E(+3) and K → E(+3) salt bridges is higher than might be expected from the observed % of salt bridges made for these pairs in the PDB (Fig. 5). These salt bridges may be underrepresented in the helices available in the PDB, which lacks high-resolution structures of SAHs. It is worth pointing out that the PDB analysis does not make any conclusions about the strength of salt bridges, as the presence of a salt bridge is defined through geometry as explained above (*i.e.* salt bridges are either present, or absent), while in MD simulations, occupancy can be used as a proxy for strength of charge–charge interaction.

Strikingly, while MD simulations showed that E–R salt bridges were more highly occupied, their average lifetimes were shorter than those for E–K (Fig. 6d, Supplementary Table S3). For example, the average lifetime for all E → R(+3) salt bridges was only 30 ps compared to 159 ps for E → K(+3) when averaged over all sequences. However, the number of E → R(+3) salt bridge formation events was 10 times higher than for E → K(+3) (Supplementary Table S3), accounting for the higher occupancy of E–R pairs. Simulations performed on the SAH from myosin-6 (M6WT) and its Lys- and Arg-only mutants (M6K and M6R) gave very similar results to those of the *de novo* sequences in terms of salt-bridge occupancies and lifetimes (Supplementary Table S4).

**Figure 7:**
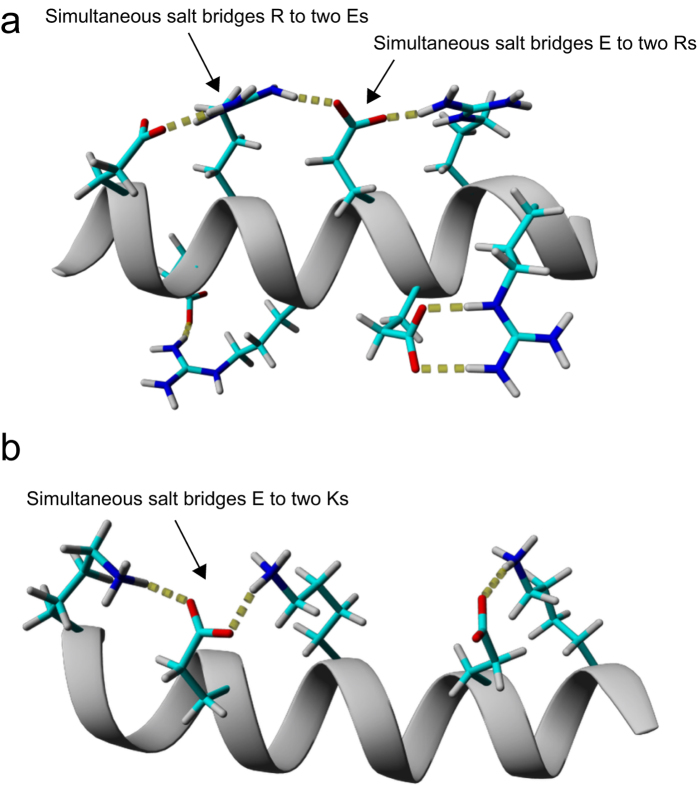
Salt bridge network formation in ER3 and EK3. Example snapshots from simulations of ER3 (**a**) and EK3 (**b**) displaying salt bridges between selected residue side chains.

Simulations also show that simultaneous salt bridges involving Arg (particularly “E–R–E” networks) form more frequently than Lys (Fig. 7, Supplementary Table S5). This may also help to explain the higher contribution to stability that Arg provides. Arg simultaneously forms salt bridges in both directions along the helix (R → E(−4) & E(+3) or R → E(−3) & E(+4) combinations, *i.e*. “E–R–E”) up to 10% of the time (Supplementary Table S5). In contrast, Lys only interacts with two Glu residues that are both C-terminal to the Lys (K → E(+3) & E(+4)) (Supplementary Table S5, not shown in Fig. 7). Other simultaneous salt bridges (X → E(−4) & E(+4) and X → E(−3) & E(+3), for X = K or R) were not significantly populated, in agreement with the experimental finding of non-cooperativity in an alanine-based peptide with a K → E(−4) & E(+4) triplet pattern^18^. Glu residues are also able to form simultaneous salt bridges with two Lys or Arg partners (Supplementary Table S4). With Lys, only those using the E → K(−4) & K(+3) combination formed (7–10% in EK3, EK2R1 and EK1R2). However, with Arg these salt bridges tended to form in the same direction along the helix (~10% for both E → R(−4) & R(−3) and E → R(+3) & R(+4)), although the E → R(−4) & R(+3) combination was also populated (~5%). Intermediate occupancies were observed for simultaneous salt bridges of Glu to one Arg and one Lys in EK2R1 and EK1R2.

Overall, MD simulations show that E–R salt bridges form more frequently than E–K salt bridges but have significantly shorter lifetimes. E–R salt bridges are more varied in terms of conformational freedom than E–K salt bridges. Additionally, simultaneous salt bridges involving Arg (particularly “E–R–E” networks) tend to form more frequently than those involving Lys.

## Discussion

Here we have successfully designed, and tested a range of polypeptides to determine the relative contributions of Lys and Arg to the stability of SAHs. We determined that: (i) the only *de novo* polypeptides to exhibit the behaviour typical of SAHs were EK3 and EK2R1, with the inclusion of a single Arg residue in EK2R1 increasing helicity and stability; (ii) any further increase in the Arg content in the *de novo* polypeptides (EK1R2 and ER3) promoted aggregation; and (iii) substituting all the Lys residues with Arg, in the naturally occurring myosin-6 SAH increased helicity and stability with respect to thermal unfolding, while substituting Arg with Lys, had the opposite effect. Thus, Lys and Arg are not completely interchangeable but make distinct and significant contributions to the stability of SAHs.

The PDB analysis and MD simulations rationalize these results. In general, they reveal that E–R pairs are more likely to form salt bridges than E–K, by utilizing multiple rotamer conformations and more binding ‘modes’, but that the lifetimes of E–R salt bridges are shorter. The most stabilizing E–R pairing (E → R(+3)) uses a wider range of side chain combinations than those for the most stabilizing E–K pairing (K → E(+4)), (*i.e.* fewer side chain rotamers need to be fixed to form salt bridge interactions for E → R(+3)). This demonstrates that the multiple binding modes of E–R pairs are separated by marginal energy barriers and they rapidly interconvert, suggesting an important entropic contribution to the stability of the helical state. For E–K, salt bridges formed by K → E(+4) pairs use fewer side-chain rotamer combinations than other pairings, and these better-defined K → E(+4) salt bridges contribute a larger favourable enthalpy to the free-energy of helix formation^17^. Thus, EK2R1, in which a single Lys is substituted by Arg in each 7 residue repeat (or substituting in Arg for Lys in the SAH from myosin-6), results in a more helical and thermally stable SAH than EK3 (or M6WT). This is important, as the ratio of K:R in naturally occurring SAHs varies^2^. Therefore, the relative proportions of K and R in these domains are likely to have biological relevance, especially given the possible variety of functions for SAHs in biological systems^2^.

The more promiscuous nature of individual E–R salt bridges also helps to explain why increasing levels of Arg in *de novo* polypeptides increased their tendency to aggregate. Shorter salt bridge lifetimes, as well as the multi-dentate nature of the guanidinium group, are likely to increase the chances that E–R pairings will form between molecules, and not just along the helix of a single molecule, explaining the tendency for *de novo* polypeptides with high levels of Arg to aggregate. Moreover, the guanidinyl group of Arg can exhibit weak hydration^26^, there can be significant pairing between guanidine-terminated side chains in polyarginine (but not between amine-terminated side chains in polylysine)^27^, and the Arg side chain can be hydrophobic above and below the plane of the guanidinyl group, allowing the stacking of Arg residues^28^. Thus, Arg side chains may stack within or between proteins, promoting oligomerization^29^. These considerations will be important for the future design of *de novo* helical polypeptides. While Arg can increase the stability of SAHs, extensive Arg “patches” in long polypeptides should be avoided, at least in designs with a regular 7-residue repeat as used herein.

Reducing the number of charged residues per repeat unit, and replacing them with Ala to maintain a high helical propensity, was not successful for generating *de novo* SAHs. Exchanging pairs of charged residues in EK3 for alanine results in an amphipathic helix pattern, with a predominantly alanine-based face, which is likely to promote protein–protein association mediated by hydrophobic contacts^22^. Not surprisingly then, the majority of these *de novo* polypeptides formed multimeric helical complexes. Alacoils are naturally occurring anti-parallel coiled coils in which alanine is the predominant residue in either the ‘a’ or ‘d’ positions of the heptad sequence repeat, and the two helical strands are closely spaced compared to other coiled coils such as leucine zippers^30^. We suspect that the hydrophobic seam formed by alanine in EK2 results in a dimer structure such as this, its stability enhanced by inter- and/or intra-chain charged interactions outside the hydrophobic seam.

The MD simulations have shown that modelled SAHs (known to be helical experimentally) are kinetically (very) stable. Such remarkable kinetic stability makes it currently unviable to explore equilibrium properties of these sequences computationally, particularly using fully solvated models. Thus, here we have focused on the contributions of K versus R to their helical state, and do not consider the effect of substituting K with R on the unfolded state. However, we would argue that the unfolded state is not relevant for our interpretation. Unfolded states are likely to be dominated by expanded coil structures due to the high charge content and charge distribution pattern of the sequence^31^. Repulsion between like-charge residues will limit the accessible conformation space available to the unfolded state and thus limit the entropic benefit of unfolding. Despite this, there will exist an expanded range of possible stabilizing salt bridge interactions in unfolded, non-helical structures, with Arg again interacting more dynamically than Lys. However, we would argue that helical forms (Arg-rich sequences in particular) benefit more through positioning their side chains to avoid charge–charge repulsion, and in their ability to dynamically rearrange and make multiple simultaneous salt bridge pairings without the need for concurrent rearrangements of the backbone. This avoids the need for disruption to the hydrogen-bonded network that makes up the core of the helix, or greater solvent exposure of hydrophobic methylene groups.

The simple repeating sequences used in EK3 and EK2R1 allows them to be easily customized for many potential synthetic biology applications. These domains have the potential to be used as force sensors, helical spacers (*i.e*. inserted between two protein domains), and/or to modulate and report on protein–protein interactions in both *in vitro* and *in vivo* applications (as reviewed in ref. 3). Choosing Lys, or a mixture of Lys and Arg subtly alters stability to provide flexibility in design. Moreover, *de novo* polypeptides can be engineered to be any length, and Ala can be replaced with other residues (*e.g*. cysteine to allow fluorescent labelling).

In summary, we have designed, expressed and characterized long, highly stable model SAHs (‘*de novo* polypeptides’), using just four amino acids, Ala, Glu, Lys and Arg. These simple designs have enabled us to gain significant insight into the mechanism by which these domains are stabilized. We have discovered Lys and Arg are not completely interchangeable, in that E–R pairings are more likely to form salt bridges than E–K thus increasing the stability of SAHs, and that E–R salt bridges are more dynamic but their promiscuous nature could contribute to the aggregation of SAHs, when Arg is increased to high levels. These data suggest that naturally occurring SAHs are likely to have different properties, depending on their relative K:R content, and provide guidelines for engineering long customizable SAHs.

## Materials and Methods

### Expression constructs

DNA sequences encoding model SAH domains were synthesized (GeneArt; GenScript) and subcloned into the pET28a SUMO vector (received as a kind gift from Dr Thomas Edwards) to introduce an N-terminal His-tag and SUMO fusion protein for increased expression and solubility as described^5^. Full sequences for these *de novo* polypeptides are provided (Fig. 1a). Each sequence contains an additional N-terminal serine residue carried over as a result of SUMO cleavage and a C-terminal tryptophan residue for UV absorbance concentration measurements. The full sequence for the SAH domain from myosin-6 (human, Uniprot ID Q9UM54, residues 926–1022) together with the mutants generated (in which Lys is substituted for Arg, and *vice versa*) is provided (Fig. 4).

### Protein expression and purification

All proteins were expressed in *Escherichia coli* BL21 Rosetta 2 (Novagen) and purified using a Ni-NTA (cOmplete His-Tag Resin, Roche) affinity chromatography column. Bacterial pellets were re-suspended in ~10 ml of buffer A (300 mM NaCl, 50 mM NaH_2_PO_4_, 10 mM imidazole, 0.1% Tween-20, 1 mM EDTA, 0.2 mM PMSF, 0.03% NaN_3_, pH 8.0 with NaOH) and sonicated on ice (Sonics Vibra-Cell sonicator, 50% amplitude, 6 cycles: 10 s on/off). Lysates were centrifuged (30,000 *g*, 20 min, 4** **°C) and supernatants applied on pre-equilibrated gravity-flow columns (1 ml of resin). Columns were washed with 50 ml of buffer B (300 mM NaCl, 50 mM NaH_2_PO_4_, 20 mM imidazole, 0.1% Tween-20, 1 mM EDTA, 0.2 mM PMSF, pH 8.0). Proteins were eluted in 8 × 1 ml fractions in buffer C (300 mM NaCl, 50 mM NaH_2_PO_4_, 200 mM imidazole, 0.03% NaN_3_, 0.2 mM PMSF, pH 8.0) and analysed by SDS-PAGE (12% gels). Proteins were then dialyzed against 150 mM NaCl (300 mM in the case of EK1), 20 mM Tris-HCl, 1 mM DTT, pH 8.0 and proteolysed for 2 h at room temperature, using ULP1 recombinant SUMO protease in a substrate to enzyme ratio 100:1. SUMO protease is a recombinant fragment of ULP1 (Ubiquitin-like-specific protease 1) from *Saccharomyces cerevisiae*. It is highly specific for the SUMO fusion protein, recognizing the tertiary structure of SUMO rather than an amino acid sequence^32^,^33^. EK3, EK2R1, EK1R2 and EK2 were separated from SUMO on 5 ml Q sepharose columns using an AKTA system. Buffers used were: 20 mM Tris-HCl, pH 8.0, 0.03% NaN_3_ (Buffer A); 1 M NaCl, 20 mM Tris-HCl, pH 8.0, 0.03% NaN_3_ (Buffer B); salt gradient: 100–600 mM. The purest fractions were combined and concentrated resulting in a 1–2 mg/ml protein solution and a typical yield of 2.5–5 mg per litre of *E. coli* culture. Purified protein was dialyzed against 100 mM NaCl, sodium phosphate (7.7 mM Na_2_HPO_4_/2.3 mM NaH_2_PO_4_), pH 7.4, and snap-frozen in liquid nitrogen for long term storage at −80 °C. An alternative method of purification was used for ER3 and EK1, which showed a high level of aggregation upon removal of SUMO. Pellets of aggregated proteins were washed 3x with 300 mM NaCl, 20 mM Tris-HCl, pH 8.0 and re-suspended in 100 mM NaCl, 10 mM sodium citrate, pH 3.5 (ER3) or 10 mM NaCl, 5 mM Tris-HCl, pH 7.4 (EK1) and dialyzed. Due to the high level of aggregation we avoided freezing these proteins and only fresh preparations were used. For CD experiments at different pH, the following buffer solutions were used – pH 2.4: 100 mM NaCl, 10 mM glycine-HCl; pH 3.5 and pH 5.0: 100 mM NaCl, 10 mM sodium citrate/citric acid; pH 10: 100 mM NaCl, 10 mM Tris; pH 12: 100 mM NaCl, 10 mM Na_2_HPO_4_-NaOH. The 15 heptad (15 H CC) and 11 heptad (11 H CC) coiled-coil fragments from human β-cardiac myosin-2 tail were expressed and purified as described previously^34^. Protein concentration was measured by absorption at 280 nm. Absorption coefficients were obtained from ProtParam software (http://web.expasy.org/protparam/). Protein concentrations used were in the range 10–40 μM.

### CD spectroscopy

CD measurements were performed on an Applied Photo Physics Chirascan CD spectropolarimeter with a 0.1 cm path length quartz cuvette in buffers as specified in the Protein expression and purification section. Data were collected every 1 nm with a scan rate of 120 nm/min; for each measurement two scans were recorded. Data presented are averaged from at least two separate measurements of different protein preparations. Thermal measurements were performed in a temperature range from 10 to 85 °C with a 0.7 °C/min heating rate, data acquisition every 1 °C. The mean residue molar ellipticity (MRE) of proteins was calculated as described^35^. Here we use the units of deg × cm^2^ × dmol^-1^, rather than the units deg × cm² × dmol^-1^ res^-1^. The helical content of proteins was calculated from values of the amide n → π* transition at 222 nm ([MRE_222_]), as previously described^35^.

### Size exclusion chromatography

Size exclusion chromatography was used to estimate the shape of the *de novo* polypeptides, and in particular to determine how elongated they were^36^. This technique separates molecules on the basis of their molecular size, and the time it takes for these molecules to elute from the column is inversely correlated with their equivalent hydrodynamic radius (Stokes radius, *R*_s_)^37^. The *R*_s_ for an elongated protein is larger than that for a globular protein of the same mass and hence the elongated proteins elute earlier from the column.

A GE Healthcare Tricorn 10/20 column was packed with Superdex 75 resin and calibrated using the GE Healthcare gel filtration calibration kit, which comprises albumin (75 kDa), ovalbumin (43 kDa), carbonic anhydrase (29 kDa), ribonuclease A (13.7 kDa) and aprotinin (6.5 kDa). The elution profiles of the *de novo* polypeptides of interest were obtained by injecting 200 μl of protein sample within a concentration range of 20–40 μM in column buffer (150 mM NaCl, 10 mM sodium phosphate, 0.03% NaN_3_, pH 7.4) onto the column at a flow rate of 0.5 ml/min, using an AKTA system. The column exclusion volume was 6.3 ml (obtained using dextran blue).

### Analytical ultracentrifugation

Sedimentation-equilibrium experiments by analytical ultracentrifugation were performed in triplicate using a Beckman Optima XL-A analytical ultracentrifuge at 20 °C with an AN50 8-place rotor, and cells with epon 6-channel centrepieces and quartz windows. Samples were prepared in 100 mM NaCl, 7.7 mM Na_2_HPO_4_, 2.3 mM NaH_2_PO_4_ and 4.61 mM NaN_3_. The samples were centrifuged in the speed range 18,000–42,000 rpm and data collected in increments of 4,000 rpm. Data were fitted to single ideal species using Ultrascan II^38^ and the confidence limits obtained by Monte Carlo analysis of the fits. Representative data for one channel for each sample are shown.

### E–R pairings in the PDB

These data were culled from the same helix dataset as used previously for E–K pairings, and analysed in an analogous manner^17^. The dataset contains helices of 12 amino acids or longer detected among 2,775 sub-1.6 Å resolution X-ray crystal structures. Interactions involving any of the first four residues of each helix are classed as ‘N-terminal’; those just involving residues at least four positions in sequence away from the N and C termini are ‘central’ and those involving the last four residues are ‘C-terminal’. The numbers of R → E(+4), R → E(+3), E → R(+3) and E → R(+4) pairs were identified. Expected numbers of pairs were estimated using the occurrence of each residue in the whole dataset. A salt bridge was considered to be formed for a pair if the centroid of Glu Oε1 and Oε2 atoms was <4 Å from any of the Arg Nε, NH1 or NH2 atoms.

*χ*_1_, *χ*_2_ side chain rotamer distributions for central salt bridge and non-salt bridge R → E(+4), R → E(+3), E → R(+3) and E → R(+4) pairs were categorized as follows: *t, χ* ≥ 120° or *χ* ≤ −120°; *g*^+^, 0° ≤ *χ* < 120°; *g*^−^, −120° < *χ* < 0° for Glu and Arg residues in all helices. Theoretical rotamer combinations were modelled in PyMOL (http://www.pymol.org) and salt bridge potential assigned if the centroid of Glu Oε1 and Oε2 atoms was <4 Å from any of the Arg Nε, NH1 or NH2 atoms and no atoms were closer than 2.5 Å to avoid steric clashes. Rotamer combinations were identified using Promotif^39^. The procedure for the RMSD calculation uses the multi-structure fitting algorithm in ProFit (http://www.bioinf.org.uk/software/profit/).

### Modelling

Explicit solvent modelling was performed using the CHARMM 36 force field^40^ parameters with TIP3P water. EK3, ER3, EK2R1 and EK1R2 structures were built as perfect α-helices, their N and C termini capped with acetyl (ACE) and methylamine (CT3) groups, respectively. Perfectly α-helical conformers were created by setting internal dihedral angles to Φ = −57° and Ψ = −47°. Structures were energy minimized for 1,000 steepest decent steps in vacuum using CHARMM^41^. Using VMD^42^, a 1.5 nm surround of water molecules (EK3: 15,310 water molecules; ER3: 17,350; EK2R1: 15,924; EK1R2: 13,505) and Na^+^ and Cl^−^ ions were added to neutralize the protein and give a NaCl concentration of ~150 mM. A further minimization (10,000 steps), 0–300 K heating protocol and short pre-equilibration was performed using NAMD^43^ (100,000 steps). Data are taken from 200-ns simulations run using NAMD at 300 K. The timestep used was 2 fs and trajectory frames were recorded every 500 steps. Simulations were performed in an equivalent manner for the SAH domain from myosin-6 and its K-only and R-only mutants (M6WT, M6K, M6R).

Wordom^44^ was used to analyse the simulation trajectories. The secondary structure of the protein was assigned for each timeframe using the DSSPcont criteria^45^. This was then used to calculate the helicity (or average helical fraction) of the protein overall. For the salt bridge analysis, the distance between Lys Nζ and the centroid of (Glu Oε1, Oε2) was calculated for each potential K → E(+4), K → E(+3), E → K(+3), and E → K(+4) pair, and the distances between each of the three Arg NH1/NH2/Nε and the centroid of (Glu Oε1, Oε2) were calculated for each potential R → E(+4), R → E(+3), E → R(+3), and E → R(+4) pair. As with the analysis of helices in the PDB, the definition of a salt bridge pairing at any frame of the trajectory required any of the resulting distances described to be less than 4 Å.

## Additional Information

**How to cite this article:** Wolny, M. *et al*. Characterization of long and stable *de novo* single alpha-helix domains provides novel insight into their stability. *Sci. Rep.* **7**, 44341; doi: 10.1038/srep44341 (2017).

**Publisher's note:** Springer Nature remains neutral with regard to jurisdictional claims in published maps and institutional affiliations.

## Supplementary Material

Supplementary Data
