# Supplementary material for: Characterization of long and stable de novo single alpha-helix domains provides novel insight into their stability
Source: Sci Rep. 2017 Mar 13;7:44341. doi: 10.1038/srep44341 (PMC5347031; doi:10.1038/srep44341)
Supplement: Supplementary Data [file srep44341-s1.doc]

**Supplementary Material.**

**Characterization of long and stable *de novo* single alpha-helix domains provides novel insight into their stability**

Marcin Wolny*^1^, Matthew Batchelor*^1^, Gail J. Bartlett^3^, Emily G. Baker^3^, Marta Kurzawa^1^, Peter J. Knight^1^, Lorna Dougan^2^, Derek N. Woolfson^3,4,5^, Emanuele Paci^1^, Michelle Peckham^1^

^1^Astbury Centre for Structural Molecular Biology, Faculty of Biological Sciences, University of Leeds, Leeds, LS2 9JT, UK; ^2^Astbury Centre for Structural Molecular Biology and School of Physics and Astronomy, University of Leeds, Leeds, LS2 9JT, UK; ^3^School of Chemistry, University of Bristol, Cantock's Close, Bristol, BS8 1TS, UK; ^4^School of Biochemistry, University of Bristol, Biomedical Sciences Building, Bristol, BS8 1TD, UK; ^5^BrisSynBio, University of Bristol, Life Sciences Building, Bristol, BS8 1TQ, UK.

**Supplementary Figures**

**Figure S1. Additional experimental data**

**Figure S2–S3. Results from analysis of crystal structures in the PDB**

**Figure S4–S5. Results from molecular dynamics simulations.**

**Supplementary Tables**

**Tables S1–S2. Results from analysis of crystal structures in the PDB.**

**Tables S3–S5. Results from molecular dynamics simulations.**

**
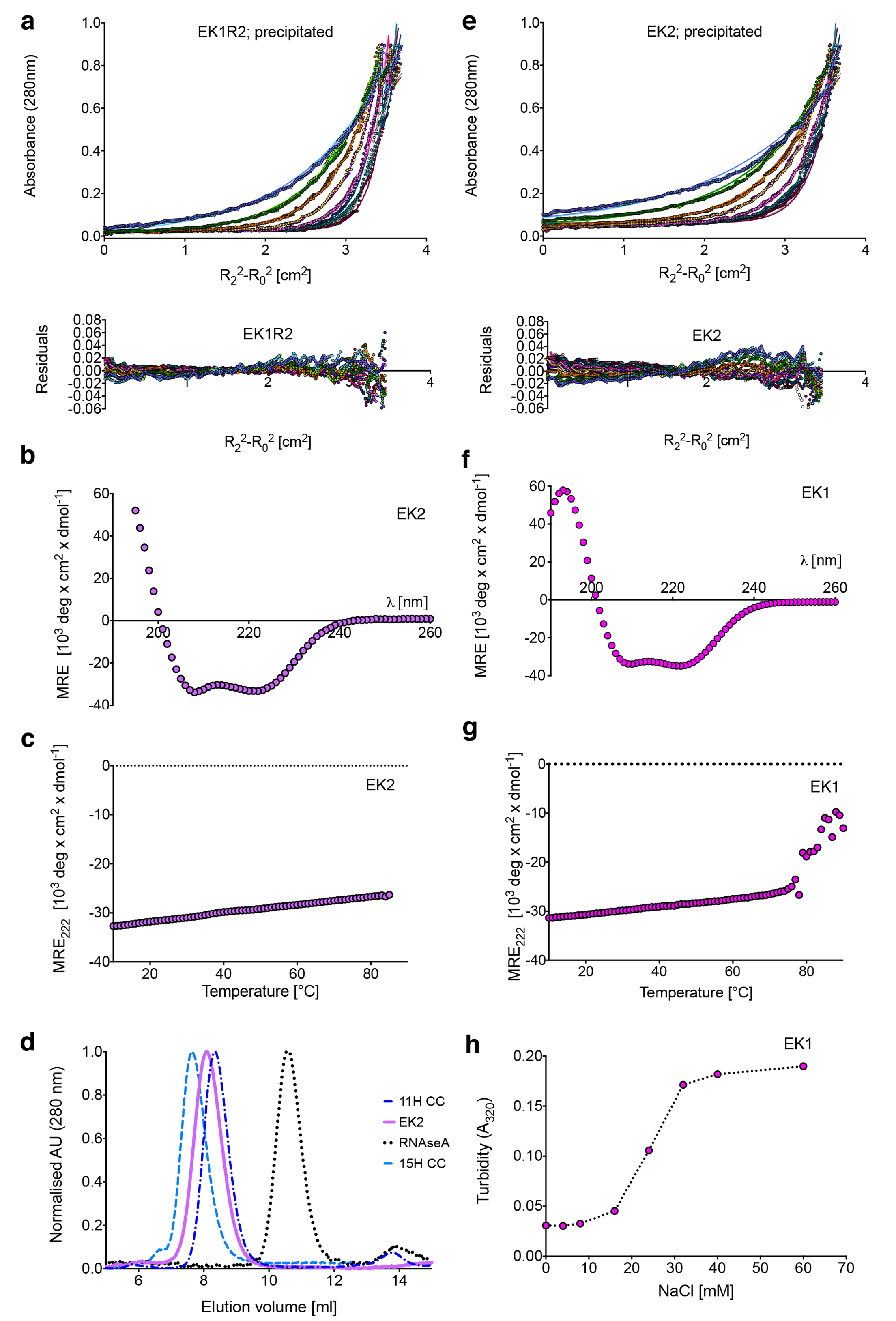
**

**Figure S1.** **Additional data for EK1R2, EK2 and EK1 peptides. (a)** EK1R2 AUC data. Top panel: data (circles) and single ideal species fits (solid lines). Lower panels: residuals of the fits. Each colour corresponds to a different speed; symbols show the raw data and lines show the fit to the data. We were unable to estimate the mass of EK1R2 due to protein precipitation. **(b)** CD spectrum (100 mM NaCl, pH 7.4, 10 °C) for EK2. **(c)** Thermal denaturation of EK2. The MRE_222_ values at each temperature are shown. **(d)** Results for gel filtration chromatography of EK2. For comparison, the results for two coiled-coil peptides from the cardiac myosin-2 tail, 15H CC (a 15 heptad construct with a dimer mass of 25 kDa), and 11H CC (an 11 heptad construct with a dimer mass of 18.3 kDa), and a globular protein of a similar molecular mass (ribonuclease A/RNAse A, 13.7 kDa) are also shown. (**e**) AUC data for EK2 (presentation as for EK1R2 in part (**a**)). We were unable to estimate the mass of EK2 due to protein precipitation. **(f)** CD spectrum (10 mM NaCl, pH 7.4, 10 °C) for EK1. **(g)** Thermal denaturation of EK1. **(h)** Solubility of EK1 (10 μM) over a range of NaCl concentrations, as measured by turbidity measurements at 320 nm. For experimental details see Materials and methods section.

**
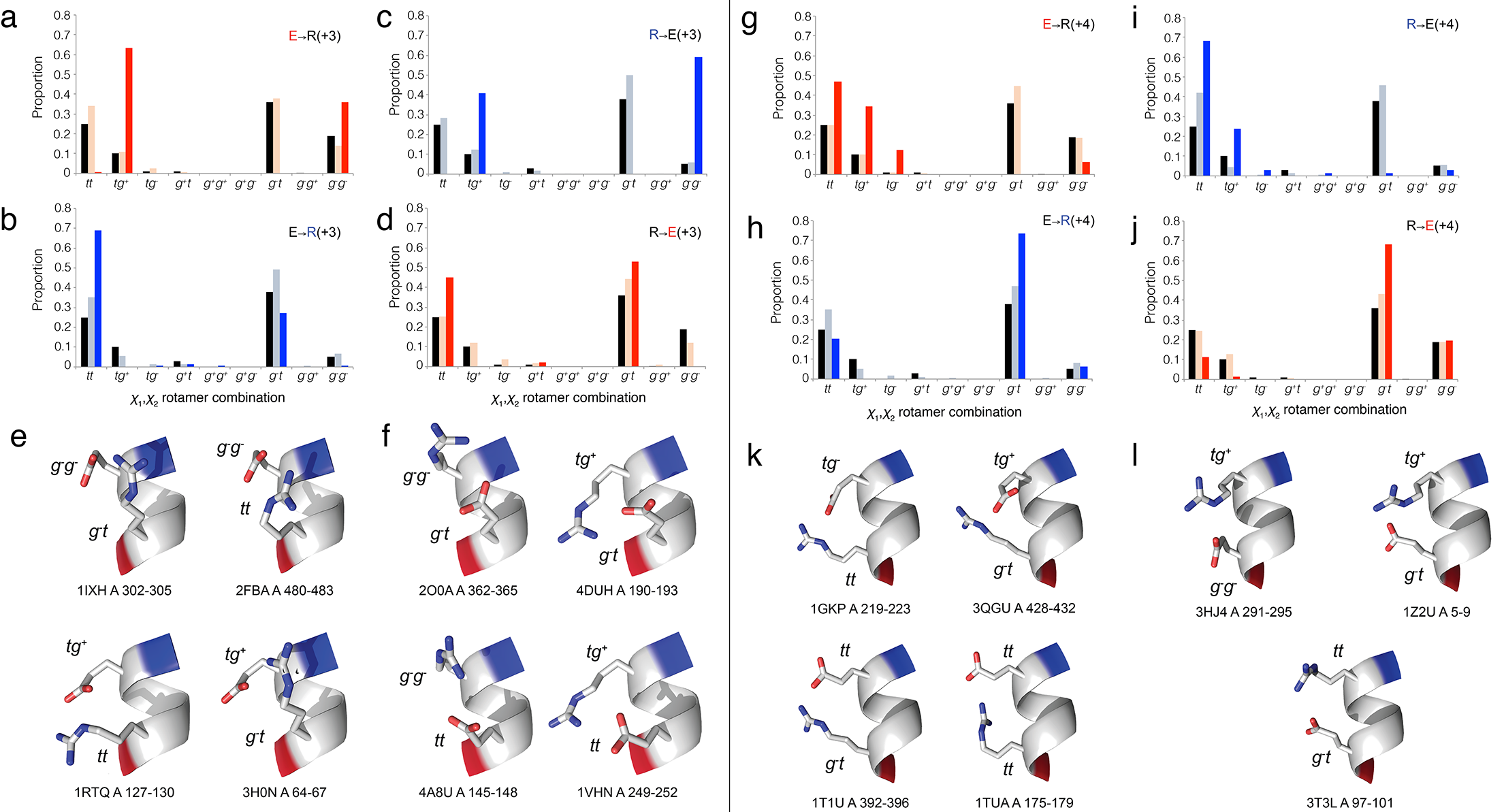
**

**Figure S2**. **Normalized frequencies of preferred *χ*_1_,*χ*_2_ rotamer combinations for (a, b) E**→**R(+3) and (c, d) R**→**E(+3) pairs for E (a, d) and R (b, c) residues and for (g, h) E**→**R(+4) and (c, d) R**→**E(+4) pairs for E (g, j) and R (i, h) residues.** Red, E; blue, R; black bars, proportion that each rotamer combination is found in all α-helices; pale coloured bars, pairs where no salt bridge is made; dark coloured bars, pairs where a salt bridge is formed. **(e, f)** Four examples of rotamer combinations that form E→R(+3) and R→E(+3) salt bridges, respectively. **(k, l)** Examples of rotamer combinations that form E→R(+4) and R→E(+4) salt bridges, respectively. PDB codes, chain letters and residue numbers are shown below the structures. Sequences run from top (N-terminal) to bottom (C-terminal) in each case.


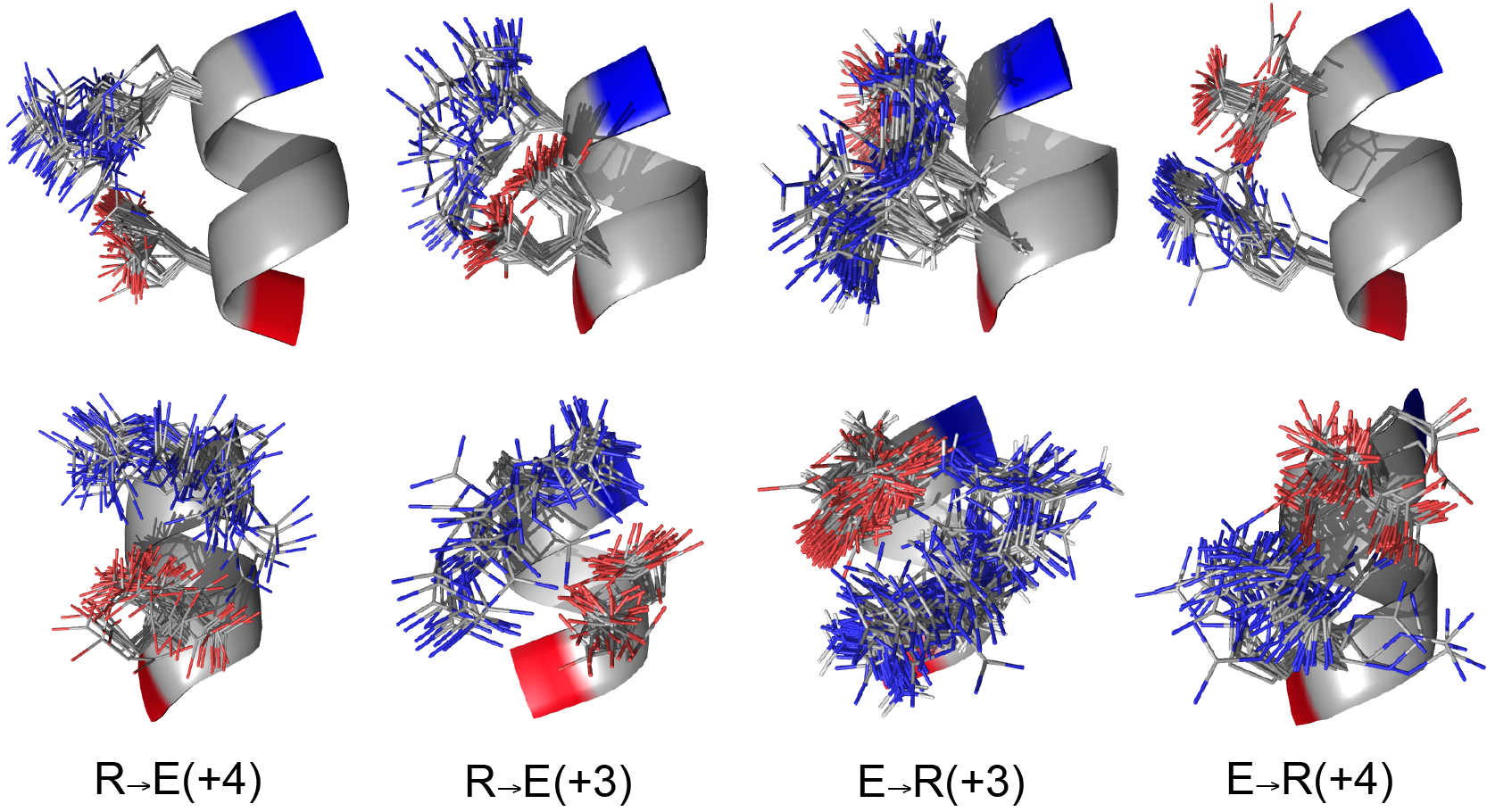


**Figure S3**. Side and front views showing overlays of ER salt bridge pairings occurring in the ‘central’ helix region. The helix runs from top (N-terminal) to bottom (C-terminal). Root mean square deviations (RMSDs) of structures from an averaged template were calculated. Mean and SD of RMSD values were: E→R(+3) 1.53 ± 0.47 Å (*n* = 133); R→E(+3) 1.41 ± 0.63 Å (*n* = 49); E→R(+4) 1.09 ± 0.56 Å (*n* = 64); R→E(+4) 1.74 ± 0.38 Å (*n* = 72). The E→R(+4) pairs show the lowest RMSD, the other RMSDs are similar.


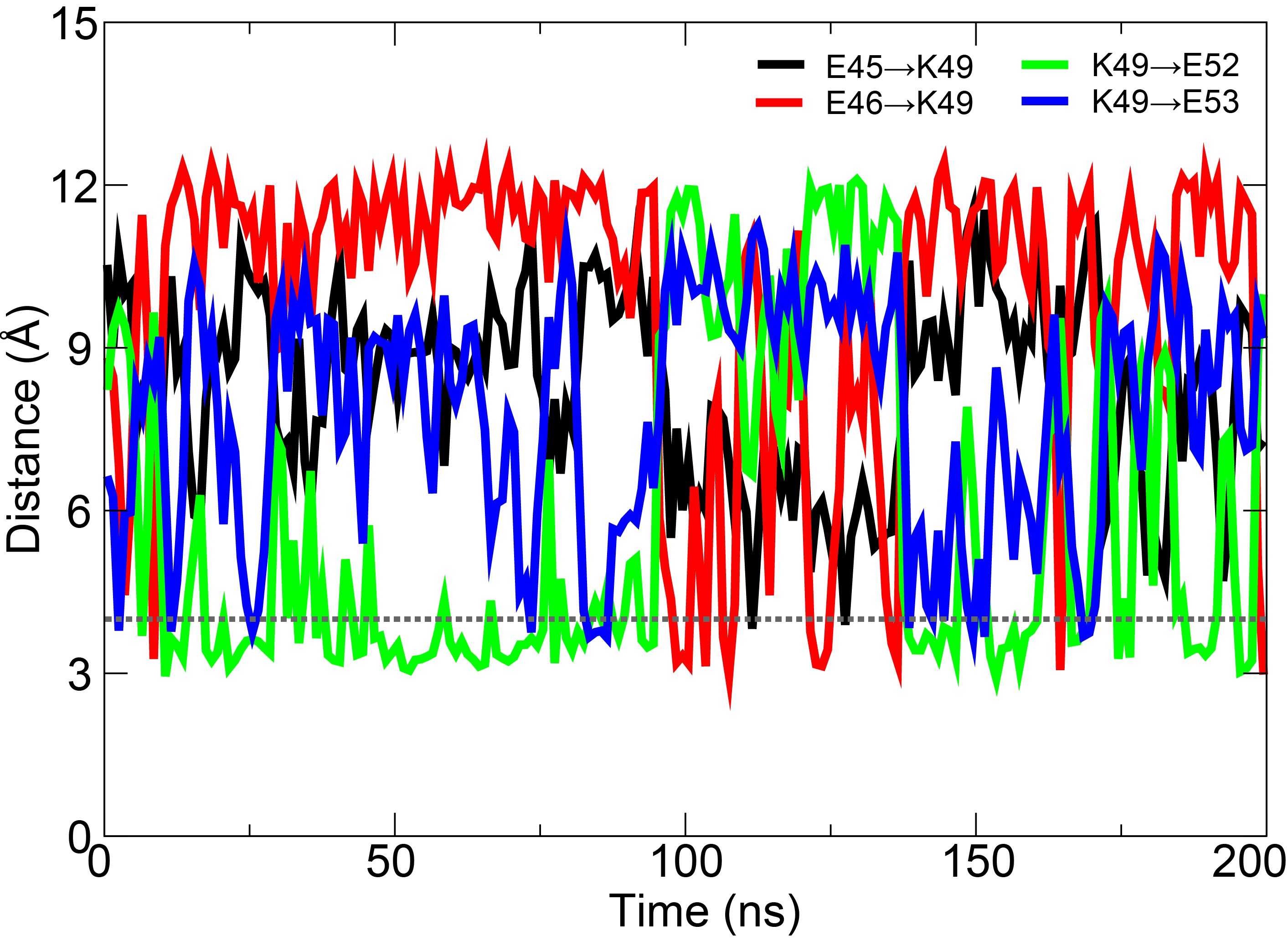


**Figure S4**. Time dependence of salt bridge pair distances for K49 in EK3. The plots are displayed as 1,000-point averages thus each point represents the distance averaged over 1 ns of simulation. The dashed line marks the 4 Å cut-off used. This line highlights a switch during the simulation from periods exhibiting mostly K→E(+3) (K49→E52, green) salt bridge formation as well as some K→E(+4) (K49→E53, blue) salt bridge formation, from 10 ns to 95 ns and 135 ns to 200 ns, to a period dominated by E→K(+3) (E46→K49, red) salt bridge formation, from 95 ns to 135 ns.

**Figure S5**. Normalized pairing distance probability distributions to neighbouring E residues for the first K in each of the AEEEKKK repeats from EK3 **(a, c)** and the equivalent R residues from ER3 **(b, d)**. **(a)** and **(b)** show the X→E(+4) pairings while **(c)** and **(d)** show E→X(+3) pairings. Note: only X→E(+4) and E→X(+3) pairings are possible from this position in the repeat. **(e, f)** Example results after grouping equivalent pairings together: normalized averaged X→E(+4) pairing distance distributions for the first, second and third X residues in each AEEEXXX repeat from EK3 (X = K) **(e)** and ER3 (X = R) **(f)**. Plots are generated from histograms of salt bridge distances using 0.1 Å bins. The figure shows that the peak positions for certain pairing distances are generally well conserved across each of the 7-residue repeats, although peak heights (*i*.*e.* probabilities for certain distances) vary between different repeats. One exception is R→E(+4), for which more significant variability is observed in both peak height and peak position beyond 4 Å. Differences in peak heights could arise from some heterogeneity in pairing populations due to the location of the repeat within the sequence. In particular, it would not be surprising for the N- and C-terminal repeats to be distinct from the central repeats, although the data do not show much evidence for this. Distributions for individual pairings are non-converged within the 200 ns timeframe, as exemplified by the limited numbers of large transitions between close contacts and well separated pairs (*e.g*. in Fig. S4). However, when individual pairings are grouped together, for instance, those K→E(+4) pairs involving the first, second or third K residue in all AEEEKKK repeats in EK3, the resulting distance distributions are very similar, especially below 4 Å (Fig. S5e and S5f). This suggests that, on average, pairing types (*i.e*. X→E(+4), X→E(+3), E→X(+3) and E→X(+4)) behave independently of their position in the sequence. By summing across equivalent X positions in each of the AEEEXXX repeats, convergence is reached for the behaviour of an “average” E–X pair.

**Table S1**.

| **a** | **R**→**E(+4)** | | | | |
| --- | --- | --- | --- | --- | --- |
| Helix Region | Observed in sequence | Expected in sequence | Observed/Expected | Observed in structure | % salt bridges made |
| N-terminal | 142 | 105 | 1.35 | 48 | 33.8 |
| Central | 242 | 163 | 1.48 | 72 | 29.8 |
| C-terminal | 285 | 150 | 1.90 | 74 | 26.0 |
| Total | 669 | 418 | 1.60 | 194 | 29.0 |

| **b** | **R**→**E(+3)** | | | | |
| --- | --- | --- | --- | --- | --- |
| Helix Region | Observed in sequence | Expected in sequence | Observed/Expected | Observed in structure | % salt bridges made |
| N-terminal | 123 | 100 | 1.23 | 26 | 21.1 |
| Central | 291 | 184 | 1.58 | 49 | 16.8 |
| C-terminal | 198 | 151 | 1.31 | 53 | 26.8 |
| Total | 612 | 435 | 1.41 | 128 | 20.9 |

| **c** | **E**→**R(+3)** | | | | |
| --- | --- | --- | --- | --- | --- |
| Helix Region | Observed in sequence | Expected in sequence | Observed/Expected | Observed in structure | % salt bridges made |
| N-terminal | 331 | 210 | 1.58 | 106 | 32.0 |
| Central | 300 | 167 | 1.80 | 133 | 44.3 |
| C-terminal | 256 | 149 | 1.72 | 111 | 43.4 |
| Total | 887 | 526 | 1.69 | 350 | 39.5 |

| **d** | **E**→**R(+4)** | | | | |
| --- | --- | --- | --- | --- | --- |
| Helix Region | Observed in sequence | Expected in sequence | Observed/Expected | Observed in structure | % salt bridges made |
| N-terminal | 295 | 198 | 1.49 | 86 | 29.2 |
| Central | 233 | 149 | 1.56 | 64 | 27.5 |
| C-terminal | 232 | 146 | 1.59 | 76 | 32.8 |
| Total | 760 | 493 | 1.54 | 226 | 29.7 |

**Table S1. Results from sequence and structural analysis of the Protein Data Bank**. Numbers of R→E(+4) (defined as R_i_→E_i+4_) **(a),** R→E(+3) (defined as R_i_→E_i+3_) **(b)**, E­→R(+3) (defined as E_i_→R_i+3_) **(c)** and E→R(+4) (defined as E_i_→R_i+4_) **(d)** pairs were identified. Expected numbers of pairs were estimated using the occurrence of each residue in the whole dataset. A salt bridge is considered to be formed if the centroid of Glu Oε1 and Oε2 atoms is <4 Å from any of Arg Nε, NH1 or NH2 atoms. Interactions involving any of the first four residues of each helix are classed as ‘N-terminal’; those just involving residues at least four positions in sequence away from the N and C termini are ‘Central’ and those involving the last four residues are ‘C-terminal’.


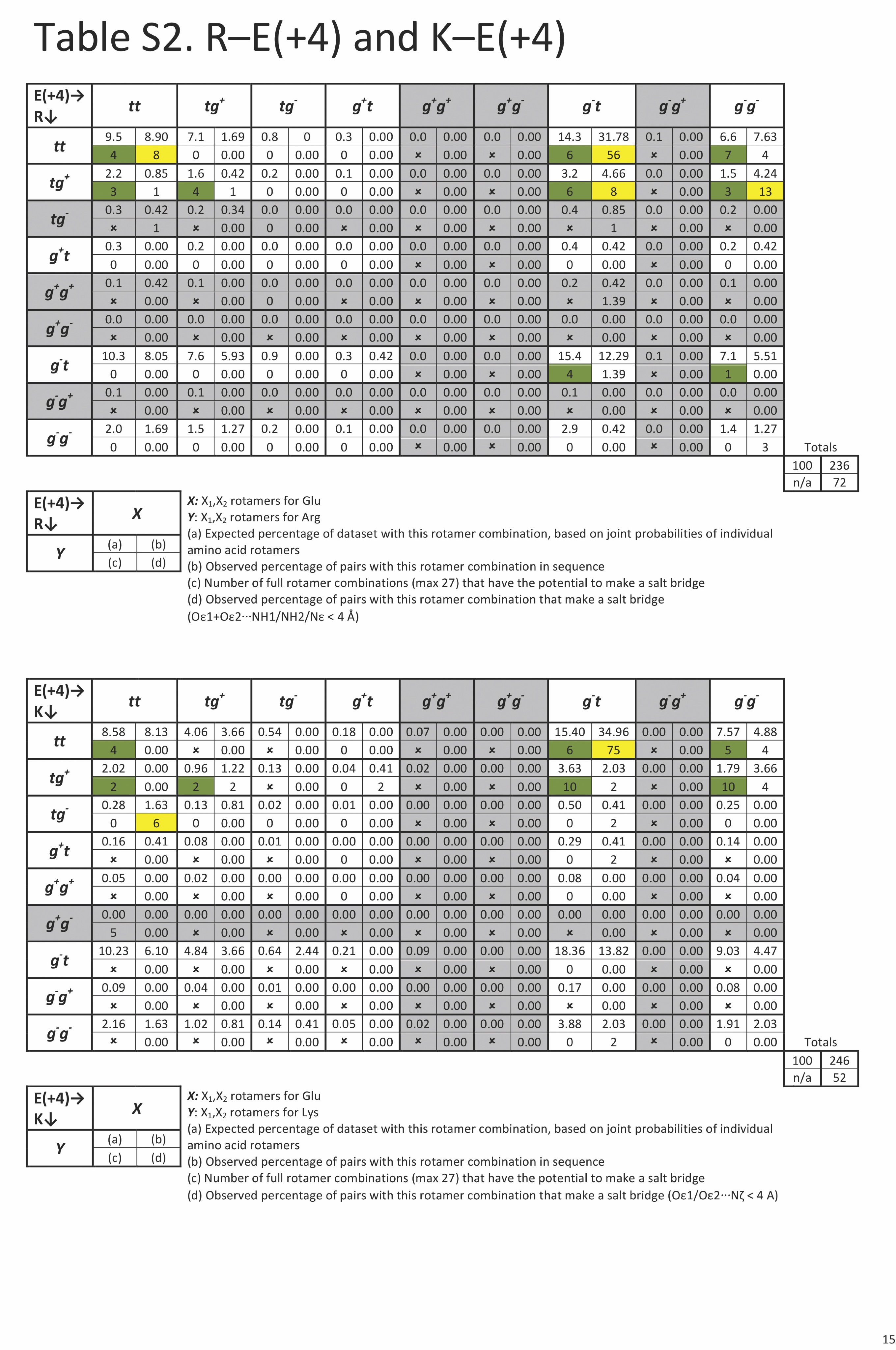

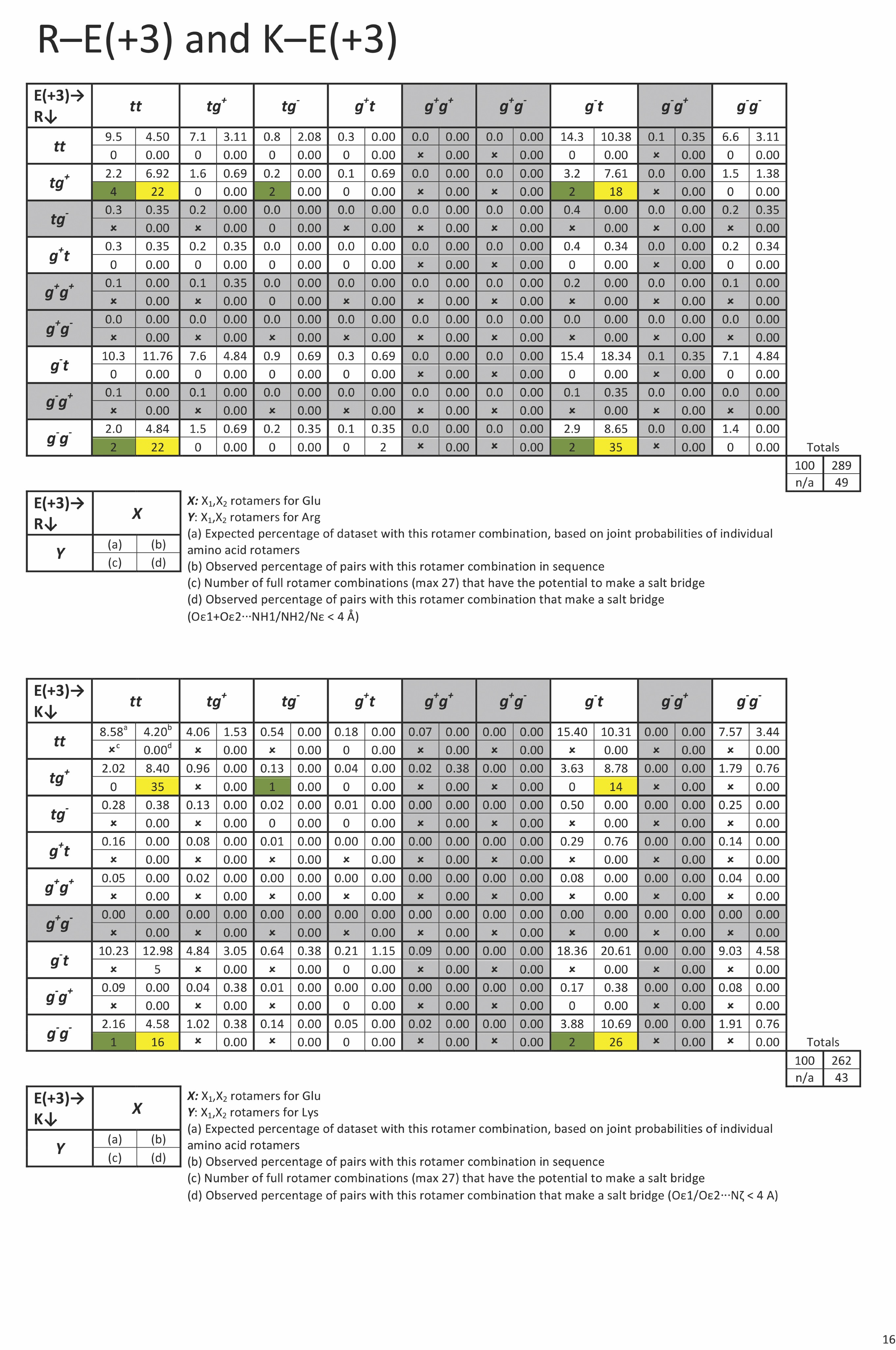

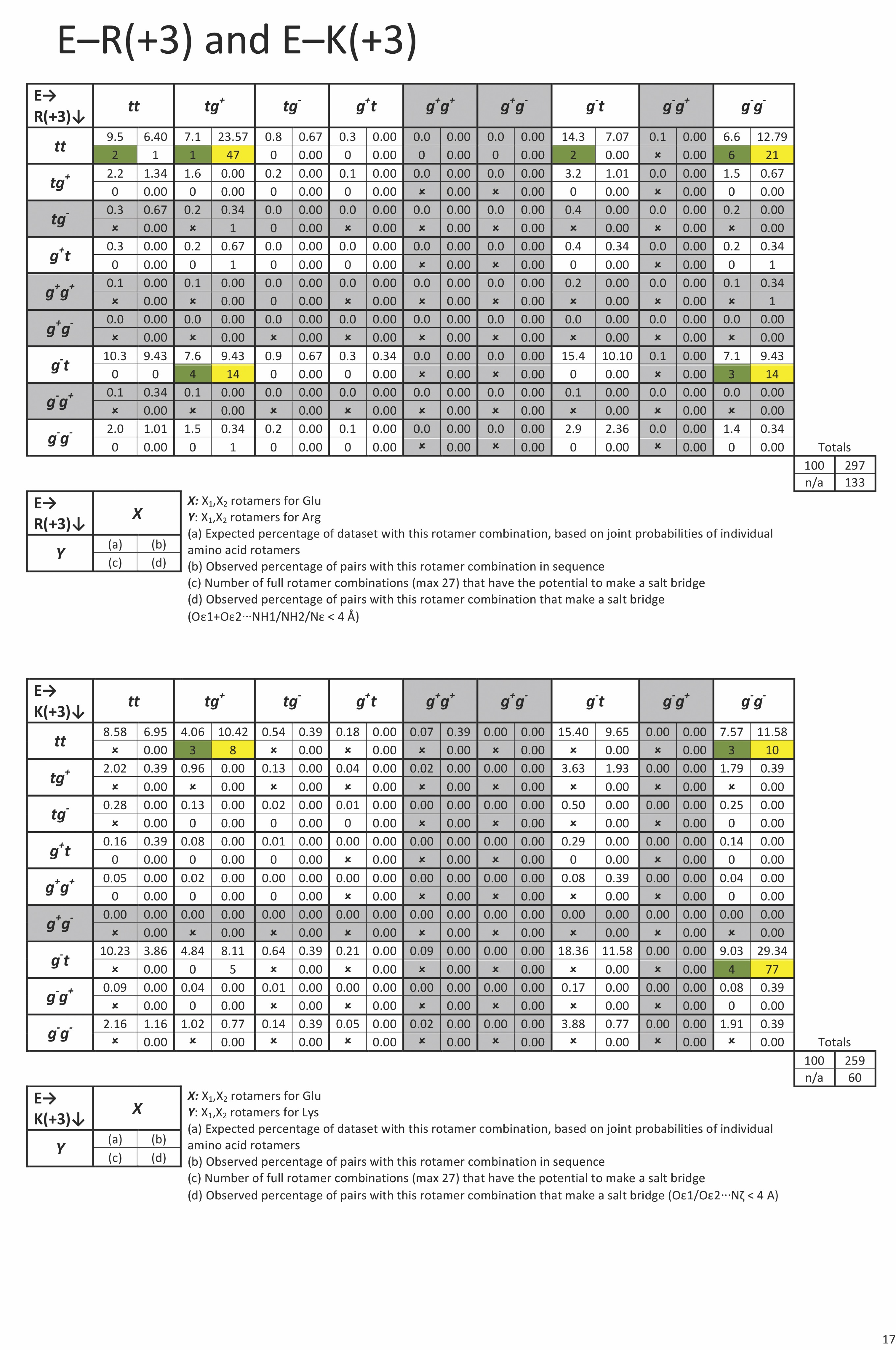

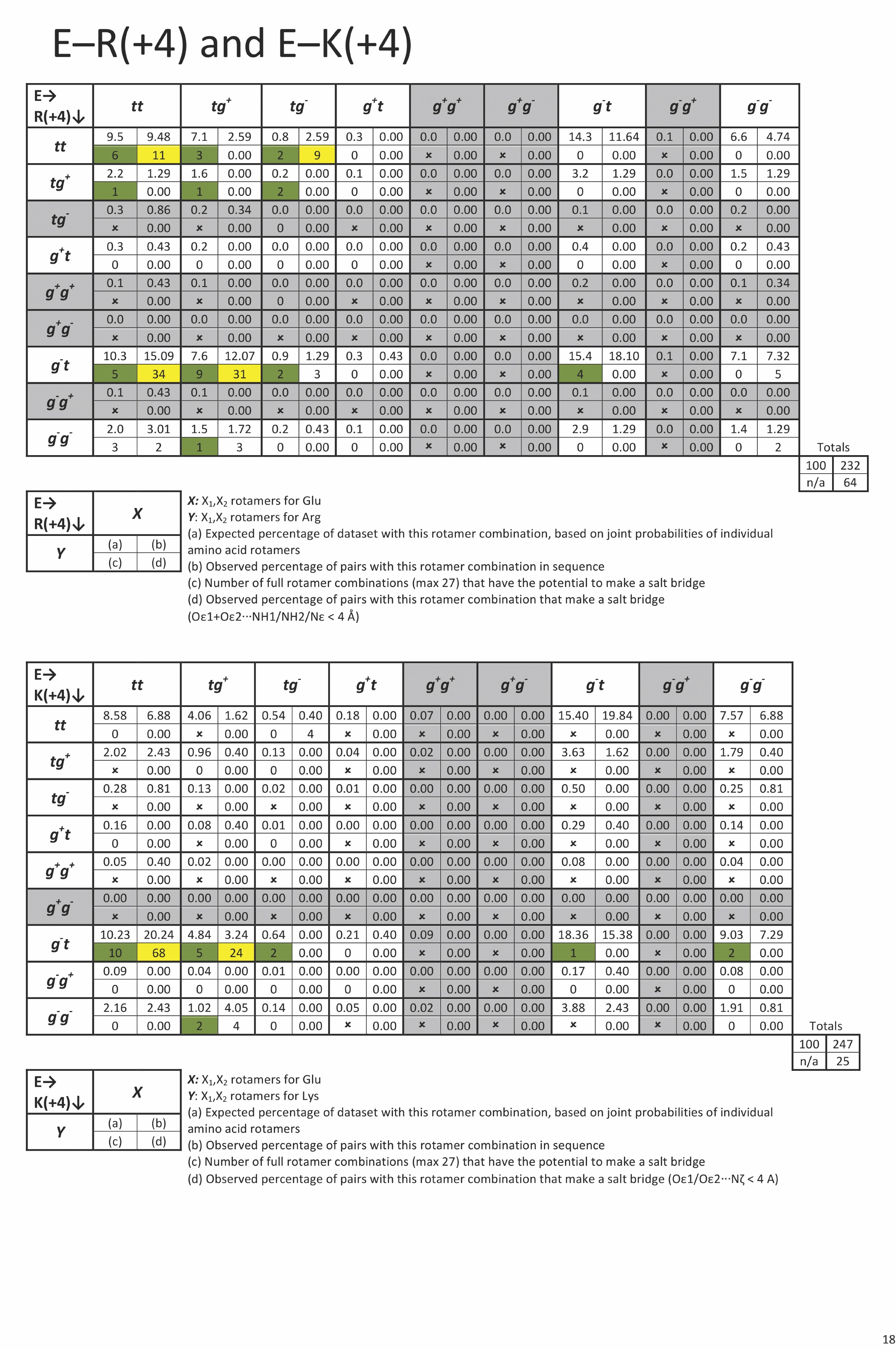


**Table S2. *χ*_1_,*χ*_2_ rotamer combinations for R**→**E(+4) and K**→**E(+4) pairs; R**→**E(+3) and K**→**E(+3) pairs; E**→**R(+3) and E**→**K(+3) pairs; and E**→**R(+4) and E**→**K(+4) pairs.** The E–K data from Baker *et al*. (2015)^1^ are shown to facilitate direct comparison. Grey shaded boxes indicate rotamer combinations that are disallowed in α⋅helices (according to Lovell *et al*.^2^) and are therefore not counted in this analysis. Green shaded boxes identify the rotamer combinations that do have the potential to form salt bridges. These were identified based on modelled AAXAAAEAA, AARAAEAA AAEAARAA and AAEAAARAA α-helices, built in PyMOL, where X was set to either R or K. The rotamers of each residue were allowed to vary according to those allowed in the backbone-dependent rotamer library provided in PyMOL, and the number of those capable of forming a salt bridge but not close enough to form a steric clash (when Oε1+Oε2⋅⋅⋅NH1/NH2/Nε < 4 Å but none of these distances < 2.5 Å) in each category recorded. The number in this box expresses the *total* number of rotamers within each *χ*_1_,*χ*_2_ category which have the potential to form salt bridges. Within each *χ*_1_,*χ*_2_ category, there are, depending on the values of *χ*_1_ and *χ*_2_, 1–3 possible rotamers for glutamate (*χ*_3_) and 1, 5, 7 or 9 possible rotamers for arginine (*χ*_3_,*χ*_4_), giving a maximum of 27 rotamers per box. Green box numbers for E–K pairs are updated from Baker *et al*. (2015)^1^ using these more stringent criteria. Yellow shaded boxes identify the rotamer combinations making salt bridges that are observed in α-helices in the PDB (>5%). Numbers (a), (b) and (d) in the table are expressed as percentages for the purposes of comparison: the total for each category is in the lower right-hand box to permit reversion back to raw numbers. Total fractions of rotamers (based on *χ*_1_,*χ*_2_ categories only) that have the potential to form a salt bridge (number of green boxes divided by *χ*_1_,*χ*_2_/*χ*_1_,*χ*_2_ categories allowed in helices) are as follows: E→R(+4) 12/30, E→K(+4) 6/48; R→E(+4) 9/30, K→E(+4) 7/48; E→R(+3) 6/30, E→K(+3) 3/48; R→E(+3) 5/30, K→E(+3) 3/48. Thus there are also fewer *χ*_1_,*χ*_2_/*χ*_1_,*χ*_2_ combinations that are likely able to result in a salt bridge for E–K compared to E–R pairings. Where there are yellow cells with no accompanying green cell, this can be attributed to unusual rotamer combinations not picked up by the PyMOL backbone-dependent rotamer library.

For E→R(+3) salt bridges, 47% adopted the *tg*^+^/*tt* (E/R) combination, utilizing the second most-preferred conformation for arginine, and a disfavoured conformation for glutamate. Other rotamer combinations *g^-^g^-^*/*tt*, *g^-^g^-^*/*g^-^t* and *tg^+^*/*g^-^t* (21%, 14% and 14%, respectively), mostly draw on the two major arginine conformations and the more-preferred glutamate conformation *g^-^g^-^*. The most dominant contribution for R→E(+3) is from *g^-^t*/*g^-^g^-^* (E/R) (35%) with arginine in one of its minor conformations and glutamate in its most-preferred conformation. Approximately 20% of salt bridges are contributed by each of *tt*/*tg^+^*, *g^-^t*/*tg^+^* and *tt*/*g^-^g^-^*, which have arginine in one of its minor conformations and glutamate in one of its most-preferred conformations. E→R(+4) pairs use the same 2 major (*g^-^t*/*tt*, 34% and *g^-^t*/*tg^+^*, 31%) and 2 minor (*tt*/*tt*, 11% and *tt*/*tg^-^*, 9%) rotamer combinations. Like K→E(+4) pairs, in R→E(+4) pairs, the dominant contribution comes from *tt*/*g^-^t* (56%), in which both residues are in one of their most-preferred conformations. Three minor contributions come from *tg*^+^/*g^-^g^-^*, *tt*/*tt* and *tg*^+^/*g^-^t* (12.5%, 8%, and 8%, respectively).

**Table S3.**

| Peptide | % occupancy (mean ± s.d) | | | | |
| --- | --- | --- | --- | --- | --- |
| **EK3** | AEEE**K**KK (1^st^) | AEEEK**K**K (2^nd^) | AEEEKK**K** (3^rd^) | All X = K | All X = R |
| K→E(+4) | 17 ± 10 | 12 ± 7 | 14 ± 7 | 14 ± 8 | — |
| K→E(+3) | — | 26 ± 6 | 33 ± 11 | 29 ± 9 | — |
| E→K(+3) | 28 ± 11 | 24 ± 10 | 27 ± 10 | 26 ± 10 | — |
| E→K(+4) | — | 4 ± 1 | 4 ± 1 | 4 ± 1 | — |
|  | | | | | |
| **EK2R1** | AEEE**K**RK (1^st^) | AEEEK**R**K (2^nd^) | AEEEKR**K** (3^rd^) | All X = K | All X = R |
| X→E(+4) | 15 ± 9 | 22 ± 14 | 13 ± 7 | 14 ± 8 | 22 ± 14 |
| X→E(+3) | — | 41 ± 17 | 28 ± 10 | 28 ± 10 | 41 ± 17 |
| E→X(+3) | 30 ± 11 | 25 ± 18 | 31 ± 10 | 30 ± 11 | 25 ± 18 |
| E→X(+4) | — | 17 ± 9 | 4 ± 2 | 4 ± 2 | 17 ± 9 |
|  | | | | | |
| **EK1R2** | AEEE**K**RR (1^st^) | AEEEK**R**R (2^nd^) | AEEEKR**R** (3^rd^) | All X = K | All X = R |
| X→E(+4) | 16 ± 8 | 13 ± 9 | 25 ± 16 | 16 ± 8 | 19 ± 14 |
| X→E(+3) | — | 35 ± 18 | 38 ± 18 | — | 37 ± 18 |
| E→X(+3) | 27 ± 11 | 31 ± 17 | 30 ± 20 | 27 ± 11 | 30 ± 18 |
| E→X(+4) | — | 20 ± 11 | 20 ± 17 | — | 20 ± 15 |
|  | | | | | |
| **ER3** | AEEE**R**RR (1^st^) | AEEER**R**R (2^nd^) | AEEERR**R** (3^rd^) | All X = K | All X = R |
| R→E(+4) | 25 ± 11 | 23 ± 16 | 25 ± 14 | — | 24 ± 14 |
| R→E(+3) | — | 32 ± 27 | 35 ± 33 | — | 34 ± 30 |
| E→R(+3) | 40 ± 23 | 31 ± 25 | 34 ± 20 | — | 35 ± 23 |
| E→R(+4) | — | 21 ± 22 | 17 ± 19 | — | 19 ± 20 |
| Peptide | Lifetimes (ps) mean ± s.d. (n=total number of events) | | | | |
| **EK3** | AEEE**K**KK (1^st^) | AEEEK**K**K (2^nd^) | AEEEKK**K** (3^rd^) | All X = K | All X = R |
| K→E(+4) | 43 ± 8 (9,937) | 45 ± 7 (7,308) | 40 ± 10 (9,277) | 43 ± 9 (26,522) | — |
| K→E(+3) | — | 89 ± 21 (8,347) | 90 ± 17 (9,870) | 89 ± 19 (18,217) | — |
| E→K(+3) | 161 ± 29 (4,634) | 178 ± 39 (3,442) | 167 ± 19 (4,102) | 169 ± 31 (12,178) | — |
| E→K(+4) | — | 11 ± 2 (9,359) | 12 ± 2 (8,216) | 12 ± 2 (17,575) | — |
|  |  |  |  |  |  |
| **EK2R1** | AEEE**K**RK (1^st^) | AEEEK**R**K (2^nd^) | AEEEKR**K** (3^rd^) | All X = K | All X = R |
| X→E(+4) | 41 ± 8 (9,470) | 8 ± 3 (116,747) | 42 ± 10 (7,723) | 42 ± 9 (17,193) | 8 ± 3 (116,747) |
| X→E(+3) | — | 52 ± 41 (43,394) | 93 ± 26 (8,614) | 93 ± 26 (8,614) | 52 ± 41 (43,394) |
| E→X(+3) | 141 ± 32 (5,831) | 35 ± 28 (34,002) | 163 ± 33 (4,927) | 152 ± 34 (10,758) | 35 ± 28 (34,002) |
| E→X(+4) | — | 7 ± 1 (81,378) | 13 ± 5 (7,974) | 13 ± 5 (7,974) | 7 ± 1 (81,378) |
|  | | | | | |
| **EK1R2** | AEEE**K**RR (1^st^) | AEEEK**R**R (2^nd^) | AEEEKR**R** (3^rd^) | All X = K | All X = R |
| X→E(+4) | 43 ± 8 (9,735) | 9 ± 6 (63,945) | 9 ± 5 (130,410) | 43 ± 8 (9,735) | 9 ± 5 (194,355) |
| X→E(+3) | — | 38 ± 26 (42,081) | 44 ± 50 (51,088) | — | 41 ± 40 (93,089) |
| E→X(+3) | 144 ± 32 (5,415) | 28 ± 22 (57,246) | 24 ± 11 (57,220) | 144 ± 32 (5,415) | 26 ± 18 (114,466) |
| E→X(+4) | — | 7 ± 2 (97,973) | 7 ± 3 (87,887) | — | 7 ± 3 (185,860) |
|  | | | | | |
| **ER3** | AEEE**R**RR (1^st^) | AEEER**R**R (2^nd^) | AEEERR**R** (3^rd^) | All X = K | All X = R |
| R→E(+4) | 8 ± 2 (111,978) | 9 ± 4 (99,196) | 7 ± 2 (118,731) | — | 8 ± 3 (329,905) |
| R→E(+3) | — | 30 ± 26 (43,751) | 43 ± 43 (30,169) | — | 37 ± 36 (151,643) |
| E→R(+3) | 39 ± 41 (49,408) | 29 ± 27 (44,984) | 23 ± 8 (57,251) | — | 30 ± 30 (151,643) |
| E→R(+4) | — | 6 ± 2 (103,371) | 6 ± 2 (76,932) | — | 6 ± 2 (180,303) |

**Table S3: Percentage occupancy and average lifetimes of salt bridge pairings made by X = R or K residues with neighbouring E residues calculated from the MD simulations.** Mean occupancies ± SD are given in red averaged for *all* potential pairings of this type over the course of the 200 ns MD simulations within EK3, ER3, EK2R1 and EK1R2. Values are also shown broken down into averages for the same position of X in the 7-residue AEEEXXX repeat (*n* = 13). Note: the final K or R in the sequence is excluded as it has only one potential salt bridge partner (E→X(+3)). Salt bridge pairings for E–K were defined as per Baker *et al*.^1^, a cut-off of <4 Å between the CoM of the Glu O atoms and the Lys side chain N atom. For E–R, salt bridges were defined using a cut-off of <4 Å between the CoM of the Glu O atoms and *any* of the side chain N atoms in Arg in E–R pairs. Mean lifetimes ± SD are shown of the average for *all* potential salt bridges of this type within EK3, ER3, EK2R1 and EK1R2 over the course of the 200 ns MD simulation. Values are also shown broken down into averages for the same position of X in the 7-residue AEEEXXX repeat. Note: the final K or R in the sequence is excluded as it has only one potential salt bridge partner (E→X(+3)). Salt bridges for each of the sidechain N atoms in Arg were counted separately. The number in parentheses indicates the total number of independent salt bridge events observed.

**Table S4.**

|  | **Mean ± SD percentage occupancy** | | **Mean Lifetimes ± SD** | |
| --- | --- | --- | --- | --- |
| **Lys** | **M6WT** | **M6K** | **M6WT** | **M6K** |
| K→E(+4) | 15 ± 6 (*n*=5) | 15 ± 7 (*n*=19) | 39 ± 18 (*n*=5, 4,988) | 43 ± 16 (*n*=19, 13,645) |
| K→E(+3) | 33 ± 27 (*n*=7) | 32 ± 23 (*n*=16) | 109 ± 115 (*n*=7, 4,295) | 74 ± 42 (*n*=16, 12,494) |
| E→K(+3) | 33 ± 29 (*n*=4) | 29 ± 15 (*n*=14) | 175 ± 44 (*n*=4, 1,348) | 149 ± 67 (*n*=14, 8,571) |
| E→K(+4) | 4 ± 3 (*n*=9) | 6 ± 3 (*n*=19) | 12 ± 5 (*n*=9, 6,788) | 11 ± 3 (*n*=19, 20,533) |
|  | **Mean ± SD percentage occupancy** | | **Mean Lifetimes ± SD** | |
| **Arg** | **M6WT** | **M6R** | **M6WT** | **M6R** |
| R→E(+4) | 25 ± 16 (*n*=14) | 24 ± 18 (*n*=19) | 11 ± 7 (*n*=14, 103,755) | 8 ± 4 (*n*=19, 194,811) |
| R→E(+3) | 38 ± 29 (*n*=9) | 45 ± 32 (*n*=16) | 22 ± 12 (*n*=9, 36,999) | 41 ± 37 (*n*=16, 51,867) |
| E→R(+3) | 37 ± 29 (*n*=10) | 29 ± 24 (*n*=14) | 39 ± 43 (*n*=10, 51,221) | 25 ± 12 (*n*=14, 50,988) |
| E→R(+4) | 28 ± 26 (*n*=10) | 17 ± 17 (*n*=19) | 8 ± 3 (*n*=10, 80,444) | 7 ± 2 (*n*=19, 129,856) |

**Table S4. Occupancy and mean lifetimes of salt bridges in myosin-6 SAH domain and the re-engineered M6K and M6R proteins.** The table shows mean ± SD percentage occupancy of salt bridges made by X = R or K residues with neighbouring E residues, averaged for all potential bridges of this type (number of potential bridges, *n*, is shown) over the course of the 200 ns simulation. E–K salt bridges were defined as per Baker *et al*.^1^, a cut-off of < 4 Å between the Centre of Mass (CoM) of the Glu O atoms and the Lys sidechain N atom. For E–R, salt bridges were defined using a cut-off of < 4 Å between the CoM of the Glu O atoms and *any* of the sidechain N atoms in Arg. The table additionally shows the mean ± SD lifetime of salt bridges made by X = R or K residues with neighbouring E residues, averaged over all potential bridges of this type (number of potential bridges, *n*, is shown) over the course of the 200 ns simulation. The second number in the parentheses indicates the total number of independent salt bridge events observed.

**Table S5**

| **Simultaneous salt bridge formation of K or R residues to two E residues (%)** | | | |
| --- | --- | --- | --- |
| **EK3** | AEEE**K**KK (1^st^) | AEEEK**K**K (2^nd^) | AEEEKK**K** (3^rd^) |
| K→E(–4) & E(+3) | — | <<1 | <<1 |
| K→E(–4) & E(+4) | — | 0 | 0 |
| K→E(–3) & E(+3) | — | 0 | <<1 |
| K→E(–3) & E(+4) | <<1 | <<1 | <<1 |
| K→E(–4) & E(–3) | — | 1 | 1 |
| K→E(+3) & E(+4) | — | 4 | 6 |
|  | | | |
| **EK2R1** | AEEE**K**RK (1^st^) | AEEEK**R**K (2^nd^) | AEEEKR**K** (3^rd^) |
| X→E(–4) & E(+3) | — | 10 | <<1 |
| X→E(–4) & E(+4) | — | 0 | 0 |
| X→E(–3) & E(+3) | — | <<1 | 0 |
| X→E(–3) & E(+4) | <<1 | 7 | <<1 |
| X→E(–4) & E(–3) | — | 2 | 1 |
| X→E(+3) & E(+4) | — | 6 | 5 |
|  | | | |
| **EK1R2** | AEEE**K**RR (1^st^) | AEEEK**R**R (2^nd^) | AEEEKR**R** (3^rd^) |
| X→E(–4) & E(+3) | — | 9 | 10 |
| X→E(–4) & E(+4) | — | <<1 | <<1 |
| X→E(–3) & E(+3) | — | <<1 | <1 |
| X→E(–3) & E(+4) | <<1 | 5 | 8 |
| X→E(–4) & E(–3) | — | 3 | 3 |
| X→E(+3) & E(+4) | — | 2 | 7 |
|  |  | | |
| **ER3** | AEEE**R**RR (1^st^) | AEEER**R**R (2^nd^) | AEEERR**R** (3^rd^) |
| R→E(–4) & E(+3) | — | 10 | 9 |
| R→E(–4) & E(+4) | — | <<1 | <<1 |
| R→E(–3) & E(+3) | — | <<1 | <1 |
| R→E(–3) & E(+4) | 9 | 9 | 9 |
| R→E(–4) & E(–3) | — | 2 | 2 |
| R→E(+3) & E(+4) | — | 5 | 6 |
| **Simultaneous salt bridge formation of E residues with two K or R residues** (%) | | | |
| **EK3** | A**E**EEKKK (1^st^) | AE**E**EKKK (2^nd^) | AEE**E**KKK (3^rd^) |
| E→K(–4) & K(+3) | 10 | 8 | 8 |
| E→K(–4) & K(+4) | <<1 | <<1 | — |
| E→K(–3) & K(+3) | <<1 | <<1 | — |
| E→K(–3) & K(+4) | 2 | 2 | — |
| E→K(–4) & K(–3) | 1 | 1 | — |
| E→K(+3) & K(+4) | <<1 | <<1 | — |
|  |  |  |  |
| **EK2R1** | A**E**EEKRK (1^st^) | AE**E**EKRK (2^nd^) | AEE**E**KRK (3^rd^) |
| E→X(–4) & X(+3) | 7 (KK) | 4 (RR) | 9 (KK) |
| E→X(–4) & X(+4) | 2 (KR) | <1 (RK) | — |
| E→X(–3) & X(+3) | <<1 (RK) | <1 (KR) | — |
| E→X(–3) & X(+4) | 2 (RR) | 2 (KK) | — |
| E→X(–4) & X(–3) | 2 (KR) | 4 (RK) | — |
| E→X(+3) & X(+4) | 7 (KR) | <1 (RK) | — |
|  |  |  |  |
| **EK1R2** | A**E**EEKRR (1^st^) | AE**E**EKRR (2^nd^) | AEE**E**KRR (3^rd^) |
| E→X(–4) & X(+3) | 8 (KK) | 3 (RR) | 5 (RR) |
| E→X(–4) & X(+4) | <1 (KR) | <1 (RR) | — |
| E→X(–3) & X(+3) | <<1 (RK) | <1 (RR) | — |
| E→X(–3) & X(+4) | 3 (RR) | 3 (RR) | — |
| E→X(–4) & X(–3) | 3 (KR) | 3 (RR) | — |
| E→X(+3) & X(+4) | 6 (KR) | 10 (RR) | — |
|  |  |  |  |
| **ER3** | A**E**EERRR (1^st^) | AE**E**ERRR (2^nd^) | AEE**E**RRR (3^rd^) |
| E→R(–4) & R(+3) | 5 | 3 | 6 |
| E→R(–4) & R(+4) | 2 | 1 | — |
| E→R(–3) & R(+3) | <1 | <1 | — |
| E→R(–3) & R(+4) | 1 | 1 | — |
| E→R(–4) & R(–3) | 8 | 7 | — |
| E→R(+3) & R(+4) | 16 | 9 | — |

**Table S5. Simultaneous salt bridge formation of K or R residues to two E residues, and simultaneous salt bridge formation of E residues with two K or R residues calculated from MD simulations.** For K or R residues to two E residues, the average percentage is shown of the total MD simulation time (for *n* = 13 equivalent positions in each 7-residue repeat) that K or R residues make simultaneous salt bridges to a combination of two of E(–4), E(–3), E(+3) and E(+4) residues, for each position over the course of the 200 ns simulation. (Note: the final K/R in the sequence is excluded as it has only one potential salt bridge partner (E→X(+3)).) Salt bridges could involve *any* of the three sidechain N atoms in Arg in E–R pairs. “<1” denotes 0.1 < *x* < 1%; “<<1” denotes *x* < 0.1%. For E residues with two K or R residues, the average percentage is shown (*n* = 12 for equivalent positions in each 7-residue repeat) of the total simulation time E residues make simultaneous salt bridges to a combination of two of X(–4), X(–3), X(+3) and X(+4) residues (X = K or R), for each position over the course of the 200 ns MD simulation. (Note: the first 3 and final 3 E residues in the sequence were excluded from analysis, as they do not have a full-set of K or R neighbours.) Salt bridges could involve *any* of the sidechain N atoms in Arg in E–R pairs.

*References*

1 Baker, E. G. *et al.* Local and macroscopic electrostatic interactions in single alpha-helices. *Nat Chem Biol* **11**, 221-228, (2015).

2 Lovell, S. C., Word, J. M., Richardson, J. S. & Richardson, D. C. The penultimate rotamer library. *Proteins* **40**, 389-408 (2000).
